# Liquid crystal–driven interfacial ordering of colloidal microplastics: Advancing microplastic characterization below the macroscale

**DOI:** 10.1126/sciadv.ady1167

**Published:** 2025-12-12

**Authors:** Fiona Mukherjee, Anye Shi, Lourdes Aoi Latasa, Fengqi You, Nicholas L. Abbott

**Affiliations:** ^1^ Smith School of Chemical and Biomolecular Engineering, Cornell University, Ithaca, NY 14853, USA.; ^2^ Systems Engineering, Cornell University, Ithaca, NY 14853, USA.

## Abstract

Detecting micrometer-scale and smaller plastic particles (microplastics or MPs) in the environment remains an unresolved challenge because they are small, have chemically heterogeneous surfaces as a result of environmental weathering, and are often accompanied by natural organic matter. Here, we advance the characterization of mixtures of MPs by leveraging their spontaneous adsorption and self-organization at liquid crystal (LC)-aqueous interfaces. We show that surface-sensitive interparticle interactions mediated by the LC can drive mixtures of colloidal MPs into assembly patterns that are accurately recognized using computer vision approaches. In particular, we show that we can identify MP composition (polystyrene and polymethyl methacrylate) in complex samples that contain natural organic matter and have been weathered using ultraviolet light. In addition, we explore how computer vision methods classify MP samples, generating fresh insights into the physical processes that control colloidal dynamics and assembly at fluid interfaces. Overall, our results advance efforts to develop characterization methods for colloidal-scale MPs that are broadly accessible (e.g., to citizen scientists).

## Teaser

Principles for screening samples for colloidal microplastics are developed using liquid crystal-aqueous interfaces.

## INTRODUCTION

Over the past two decades, awareness of the presence of microplastics (MPs) in environmental waters has increased (*1*–*3*). Substantial advances in understanding the sources, composition, distribution, and potential toxicity of the MPs have also been reported. MPs are now understood to enter aquatic environments as both primary particles (i.e., objects manufactured as micro- or nanoparticles) and secondary particles (i.e., fragments derived from the breakdown of larger plastic objects), with the most common MPs comprising polystyrene (PS), polyethylene (PE), polypropylene (PP), polyethylene terephthalate (PET), or polyacrylates (*1*). While there is extensive literature on the transport and behavior of MPs in the millimeter range, fewer studies of plastic particles in the micro- and nanometer size ranges have been reported (*4*, *5*). This situation reflects challenges associated with isolating and characterizing particles smaller than 50 μm, largely due to limitations of sieving techniques that are typically used to capture and sort MPs (*6*). An additional challenge is the presence of colloidal natural organic matter (NOM) in environmental waters, which adds multiple sample processing steps between MP capture and characterization (*7*–*9*). Small MPs, however, are reported to be more abundant than larger-size classes in freshwater sediments, with the smaller MPs staying at the water surface longer and posing a higher threat because of their increased capacity to adsorb organic toxins. This paper addresses the challenge of detection and characterization of micrometer-sized MPs. In the remaining text of this paper, we use the term MPs to refer to micrometer-sized plastic particles.

The most commonly used methods for identification of MPs involve visualization, including viewing of MPs under a microscope. When used to characterize MPs in water samples, however, the approach suffers from rates of false positive identification that range from 20 to 70% (*10*), with organic matter and inorganic species such as aluminosilicates being incorrectly identified as MPs. The rates of misidentification are even higher (~99%) when sampling complex matrices such as sediment samples (*11*). To address the high false positive rates, spectroscopic techniques such as energy dispersive x-ray spectroscopy, Raman spectroscopy (*12*), or Fourier transform infrared spectroscopy (*13*–*15*) have been used. Although spectroscopic methods comprise the current gold standards for MP characterization, the instruments used for analysis are too complex and expensive to be broadly adopted by many scientists, including citizen scientist groups who are largely responsible for the collection and characterization of MPs in inland waters and coastal regions of the US (*16*, *17*). We envisage community scientists potentially using the approach advanced here for initial screening of samples. If additional validation of chemical identity is required after initial screening, citizen scientists would send select samples to an analytical laboratory for chemical analysis using techniques such as Fourier transform infrared or Raman spectroscopy.

Here, we report the development of fundamental principles for characterization of colloidal-scale MPs based on liquid interfaces. In particular, prior studies have demonstrated that colloids can be steered along self-organization pathways at liquid-liquid interfaces in ways that depend strongly on colloid surface properties, thus leading to two-dimensional (2D) assemblies of colloids with characteristic assembly patterns (*18*–*23*). We seek to leverage that surface sensitivity into methods for characterization of MPs. Colloidal aggregation has also been studied in bulk aqueous and organic solvents, revealing how colloidal interactions influence 3D aggregation patterns (*24*, *25*), but 2D aggregation at interfaces is simpler to characterize than 3D aggregation in bulk dispersions. Accordingly, here, we focus on characterization of MPs via their 2D aggregation behaviors at fluid interfaces.

A majority of past studies of 2D aggregation of colloids at fluid interfaces have been performed at isotropic oil-water interfaces, and the aggregation patterns observed have been attributed to capillary, dipolar, electrostatic, van der Waals, and depletion forces (*24*–*29*). More recently, however, it has been demonstrated that additional colloidal interactions (beyond those present when using isotropic oils) can be introduced at liquid crystal (LC)-water interfaces, interactions that are particularly sensitive to colloidal surface properties (*30*–*35*). Although LC-mediated colloidal interactions at LC-water interfaces are not yet fully understood (see below), in bulk LC phases, the presence of a colloidal particle typically strains the LC and introduces topological defects, thereby generating both long-range (because of elastic energy arising from strain) and short-range (because of defects) interactions between neighboring colloidal particles (*36*, *37*). Such interparticle interactions in LCs are known to be sensitive to colloidal surface properties such as surface roughness, surface chemical functionality, and surface charge, leading to assemblies of colloids with organizations that reflect surface properties (*32*–*34*, *38*, *39*). Here, we explore the use of LCs to amplify subtle differences in the surface properties of MPs into distinct interparticle interactions, thus steering the MPs into recognizable aggregation patterns that reflect MP chemical identity [including chemical identity as modified by history such as ultraviolet (UV) weathering].

Past studies of colloidal interactions at LC interfaces include investigations of liquid droplets (glycerol) and solid particles (silica) (*30*, *31*, *33*, *38*, *40*). For droplets or micrometer-sized solid particles with uniform surface chemistry, gravitational forces and contact line pinning are typically insufficient to deform the LC interface; hence, capillary forces are weak compared to colloidal interactions arising from LC elasticity. Under these conditions, observations of the ordering of droplets/particles are consistent with predictions of equilibrium ordering controlled by LC-mediated interactions (e.g., glycerol droplets have been observed to form 2D crystals). In contrast, however, MPs typically have heterogeneous surfaces, including nonuniform surface chemistry (e.g., patchy charge density) and surface roughness (*25*), which leads to contact line pinning, interface deformation, and strong capillary interactions. In a past study, we found that microparticles of PS or PE exhibit distinct patterns of organization at the LC-aqueous interface (*34*), a result that was attributed to differences in surface roughness caused by the crystalline and amorphous structures of PE and PS, respectively. While other studies of colloidal particles with nonuniform surface chemistry at LC interfaces have revealed colloidal organizations that likely reflect capillary interactions (*30*, *31*, *41*, *42*), a full understanding of LC-mediated colloidal interactions at fluid interfaces remains to be established. In the current study, we provide fresh insights into the physical processes by which colloidal dynamics and nonequilibrium interfacial states influence the assembly of colloids at LC-aqueous interfaces. In particular, we connect our observations to prior experimental and simulation-based studies (*36*, *37*) that have revealed that the mobilities of colloids at LC interfaces can be influenced by the local ordering of LC around the colloids (*43*, *44*).

**Fig. 1.**
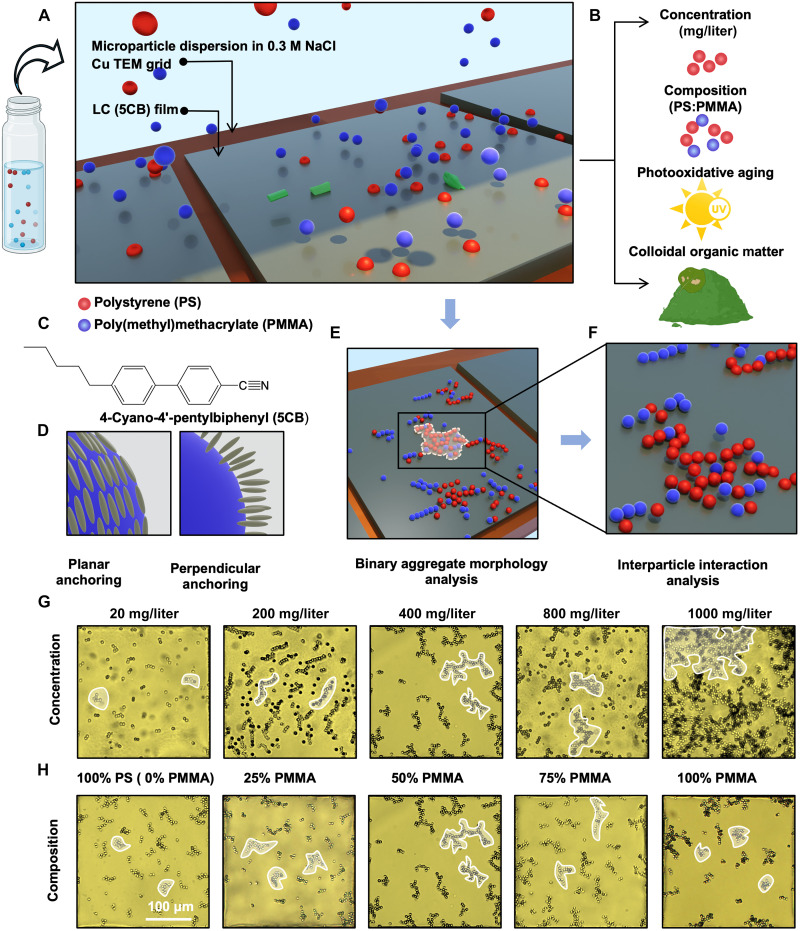
Overall framework for prediction of polymeric identity and sample parameters of MP mixtures within complex samples. (**A**) Schematic illustration of the experimental setup for collection of PS (red) and PMMA (blue) MP mixtures at a thermotropic LC (5CB)-aqueous interface. (**B**) Illustration of four degrees of freedom in sample parameters investigated. (**C**) Molecular structure of the LC (5CB) used in this study. (**D**) Schematic illustration of two predominant configurations of LC mesogen orientation on the particle surface: planar and perpendicular. (**E** and **F**) Schematic illustration of PS (red) and PMMA (blue) MP mixture assembly patterns at the LC interface, analyzed for (E) aggregate morphology and (F) internal organization of MPs within clusters under different experimental conditions. (**G**) Representative bright-field optical micrographs of a 50% PS and 50% PMMA MP mixture showing aggregation patterns for five different MP concentrations: 20, 200, 400, 800, and 1000 mg/liter. As mentioned in the main text, these concentrations are higher than environmental MP concentrations and are typical of preconcentrated samples commonly used for analysis. (**H**) Representative bright-field optical micrographs showing aggregation patterns for mixture compositions on which the composition classification problem is evaluated. The images are for the 400 mg/liter MP concentration in 0.3 M aqueous NaCl, with the following compositions in bulk aqueous dispersion: 100% PS, 75% PS-25% PMMA, 50% PS-50% PMMA, 25% PS-75% PMMA, and 100% PMMA. Note that the middle image in (G) and (H) refers to the same experimental condition and is duplicated to clearly show the evolution in aggregation of MPs with varying MP concentration (G) and PS-PMMA composition (H). All optical micrographs have the same scale. The white boundaries indicate MP aggregation patterns characteristic of each sample type. In row (G), the MP clusters grow in size from left to right, whereas, in row (H), the aggregates exhibit a greater extent of branching in the middle (mixed compositions) as compared to the two ends (single-component MP composition).

We address several key questions that underlie the behaviors of MPs at LC interfaces. First, we address the question of whether or not two MPs, both formed from amorphous polymers that generate weak capillary interactions [we use PS and polymethyl methacrylate (PMMA)], will generate distinct assembly patterns at LC-aqueous interfaces. Second, we investigate the assembly of complex mixtures of MPs that include soluble and dispersed NOM and various degrees of weathering by exposure to UV light. We ask whether aggregation patterns formed by these complex mixtures can be recognized by computer vision methods to infer the sample composition and history. Third, we report physical insights into the colloidal processes that give rise to the aggregation patterns formed by complex MP mixtures. Specifically, we ask whether the aggregation patterns of colloids at LC interfaces (illustrated in Fig. 1, E and F) can be understood within a nonequilibrium framework that considers the effects of LC ordering on both MP mobility and LC-mediated elastic interparticle forces.

## RESULTS

In our initial experiments, we probed the role of four experimental degrees of freedom on MP aggregation at LC-aqueous interfaces: (i) MP concentration, (ii) MP mixture composition, (iii) duration of aging by UV light, and (iv) presence of dispersed and soluble NOM. Below, we report findings from these studies and, subsequently, in Discussion, provide mechanistic insights into the origins of the MP aggregation behaviors.

### How are the states of aggregation of binary mixtures of PMMA and PS at LC interfaces affected by sample concentration and mixture composition?

We hypothesized that samples containing MP mixtures that differ in concentration and/or composition would generate distinct MP aggregation patterns at LC interfaces and that the aggregation patterns can be recognized by trained computer vision algorithms. Here, we provide an initial test of this hypothesis using binary mixtures of PS and PMMA. First, we describe how changes in the total MP concentration of an equimolar mixture of PS and PMMA affect interfacial aggregation. Second, we report experimental results in which we vary MP mixture composition at a fixed concentration of MPs.

Figure 1G shows representative bright-field optical micrographs of LC-aqueous interfaces after incubation against MP mixtures containing equal parts of PS and PMMA, with a total MP concentration of either 20, 200, 400, 800, or 1000 mg/liter. We selected these MP concentrations because they caused a measurable number of MPs to sediment and aggregate at the LC interface within 15 min of incubation. While the concentrations are higher than those found in the environment (1 to 10 μg/liter is common) (*45*, *46*), environmental samples are typically concentrated (e.g. by sedimentation, sieving, etc.) before analysis (*45*, *46*). Inspection of Fig. 1G (across the row) reveals that the MP mixtures spontaneously assemble into discrete, fractal-like aggregates at the LC-aqueous interface. For samples containing low MP concentrations (20 mg/liter) (Fig. 1G), we observed discrete chain-like aggregates to form, and with increasing MP concentration, elongated (200 mg/liter) and subsequently multibranched aggregates (400 and 800 mg/liter) were observed. Both the number density of MPs on the LC-aqueous interface and the average number of MPs per cluster were measured to increase with the concentration of MPs in the bulk (Fig. 1G and figs. S1 to S3). At concentrations higher than 800 mg/liter, however, we observed a qualitative change in the behavior of the MPs and their aggregates at the LC interface: Within 15 min of incubation, the MPs formed extended networks that were arrested in motion because of confinement by the boundaries of the transmission electron microscopy (TEM) grid squares (Fig. 1G). This behavior is reminiscent of a jamming transition within a 2D colloidal network (*47*, *48*). For the remainder of the study reported here, we focus on MP concentrations that yield discrete clusters (concentrations up to 800 mg/liter).

In the second set of experiments, we varied the binary MP mixture composition while keeping the total concentration of MPs fixed at 400 mg/liter. We explored five MP mixture compositions; 100% PMMA, 75% PMMA-25% PS, 50% PMMA-50% PS, 25% PMMA-75% PS, and 100% PS. Figure 1H shows a representative set of bright-field optical micrographs of MP aggregation patterns formed by the mixtures. Inspection of the fractal-like aggregation patterns across Fig. 1H hints that single-component MP samples (100% PS or 100% PMMA) form interfacial aggregates that are more compact and dense as compared to the binary mixtures. However, visual inspection also revealed substantial sample-to-sample variation for each mixture composition. This motivated us to apply computer vision–based methods to obtain statistically significant metrics of the aggregate structures and to explore whether the aggregation patterns could be reliably analyzed to indicate the composition and concentration of MPs within samples. We note here that the mixture compositions defined above correspond to MP compositions in the aqueous dispersion and not on the LC interface. We report on the compositions of aggregates formed on the LC interface below.

**Fig. 2.**
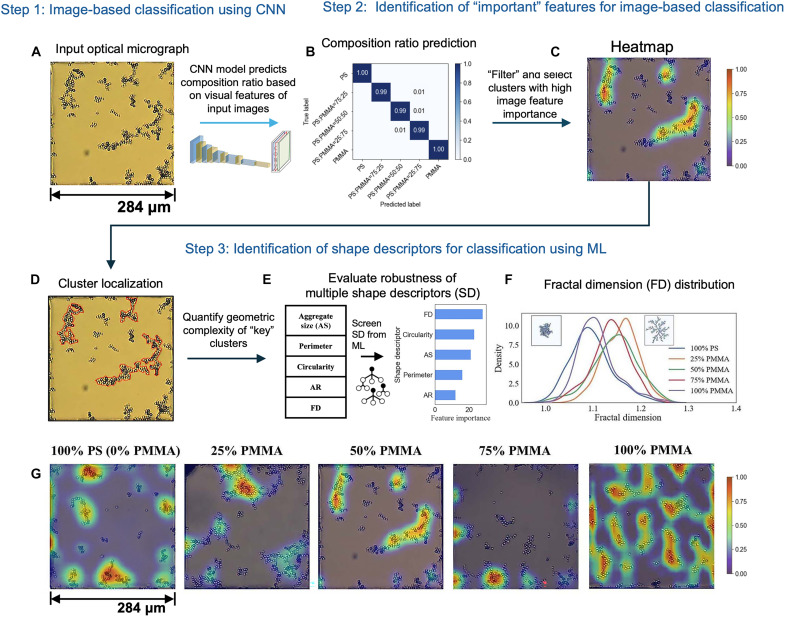
Categorizing MP mixture compositions on the basis of optical fingerprints at the LC-aqueous interface using machine learning. Schematic illustration of image identification using the proposed CNN model, DeepPolyNet, as the first step (step 1) for predicting PS-PMMA binary mixture ratios on the basis of image visual features. (**A**) Bright-field optical micrograph of one TEM grid square used as an input image for classification. (**B**) Confusion matrix of classification performance for different composition ratios of PS and PMMA mixtures. (**C**) Heatmap generated by superimposing a color map or contour plot from GradCAM++ feature importance analysis results on an input bright-field optical micrograph shown in (A). The color bar from blue to red reflects feature importance from 0 to 1. Step 2 analysis identifies the “important features” that contribute to accurate image identification. (**D**) Optical micrograph in (A) with highlighted contours of “key clusters,” identified in step 2. In step 3, only clusters with high feature importance identified in step 2 are selected for each composition class. The contours of selected clusters are localized for further downstream morphology analysis. (**E**) Evaluation of robustness of a range of shape descriptors to leverage structural differences between “key clusters” from five classes of mixture compositions. Five shape descriptors are used: aggregate size (AS), perimeter, circularity, aspect ratio (AR), and FD. The bar plot presents the relative feature importance of multiple morphological properties toward differentiating mixture composition differences. (**F**) Plot of FD distribution of “key clusters” for all five classes of mixture compositions. The plot suggests that MP mixture compositions have higher FD values than single-component samples. (**G**) Corresponding GradCAM++ heatmaps for optical micrographs showing five classes of mixture compositions on which the composition classification problem is evaluated. The images are for 400 mg/liter MP concentration in 0.3 M aqueous NaCl, with the following compositions in bulk aqueous dispersion: 100% PS, 75% PS-25% PMMA, 50% PS-50% PMMA, 25% PS-75% PMMA, and 100% PMMA. Note that the optical micrograph shown in (A) is used in (C) and (D) to show how heatmaps (C) and cluster contours (D) are calculated from the optical micrograph (A). In addition, the representative heatmap in (C) is also included in (G) to convey how the heatmaps change with the composition of the MPs.

Our computer vision–based approach to the analysis of MP aggregation patterns at LC interfaces involved three steps (Fig. 2). First, we trained a convolution neural network (CNN) called DeepPolyNet to recognize optical micrographs of aggregation patterns exhibited by the MP mixtures on the LC interface, as shown in Fig. 2 (step 1). We tested the capability of the trained CNN to identify the composition and concentration of unknown MP samples on the basis of their aggregation patterns. Second, we determined the key structural features of images used by DeepPolyNet for classification of MP sample concentration and composition (step 2). Third, motivated by the finding in step 2 that DeepPolyNet analyzed the structure of aggregates to infer MP sample concentration and composition, we identified potential descriptors of the aggregate structures (so-called shape descriptors). We then evaluated the relative effectiveness of the shape descriptors (individually or in groups) for determining MP mixture composition and concentration from MP aggregation patterns at LC interfaces. Below, we detail our findings under each of these three steps.

The dataset that we used to train DeepPolyNet (step 1 in Fig. 2A) consisted of 200 optical micrographs for each of the four MP concentrations and five MP compositions, totaling 4000 images and 20 sample types. The 200 optical micrographs used for each sample type were acquired from five independent experiments. Each independent experiment involved four TEM grids. We acquired 10 images from each TEM grid, with each image showing MP aggregation patterns in one square of the TEM grid.

We present a detailed description of the CNN used in our study in the Supplementary Materials (section SI. 4). Briefly, DeepPolyNet uses the convolutional architecture on the basis of the previously reported VGG16 model for feature extraction but introduces a global average pooling layer to reduce feature dimensions. DeepPolyNet leverages pretrained convolutional weights from the VGG16 model trained on the ImageNet dataset (*49*), applying a transfer learning strategy. While most of the convolution layers in DeepPolyNet retain the original pretrained weights of VGG16, the final (fifth) convolution block and the classifier layer are retrained using images from the MP aggregation pattern dataset. This approach seeks to maximize the ability of the model to recognize the MP aggregation patterns.

Before implementing DeepPolyNet, we evaluated a series of CNN models (Xception and EfficientNetB7) and vision transformer models for feature extraction, as discussed in the Supplementary Materials (section SI. 5), and found that the convolutional layers of VGG16 consistently delivered the best performance. VGG16’s simple sequential architecture helps prevent overfitting on small datasets, while vision transformer models generally require large datasets to perform well. In addition, VGG16 effectively captures local spatial features with limited training samples, making it more suitable for our dataset of five classes with 200 images per class.

Overall, the experimental dataset of 4000 images was used as follows: 72% for training the model, 18% for validating its performance during development, and 10% for final testing. The results of the final testing of the retrained CNN are shown in Fig. 2B as a so-called confusion matrix, which characterizes the accuracy with which DeepPolyNet is able to recognize specific sample types. Inspection of Fig. 2B reveals that the CNN is able to recognize 100% PS and 100% PMMA sample types with an accuracy of 100%. For the three mixtures of PMMA and PS, the accuracy with which the CNN can identify the mixture composition is 99%. We also evaluated the performance of DeepPolyNet for recognition of sample concentration (fig. S5), where each concentration included samples from all five different compositions. Our results show that DeepPolyNet recognizes the sample concentrations with high accuracy, with concentrations of 400 and 20 mg/liter being recognized with the highest (99.5%) and lowest (97.8%) accuracy, respectively.

In step 2 of our computer vision–based analysis (Fig. 2C, step 2), our goal was to identify the key features of the images of aggregated MPs used by DeepPolyNet to assign the MP composition and concentration. We used the algorithm called GradCAM++ (*50*) to create a heatmap that indicates the relative importance of visual features in each image that contribute to classification. GradCAM++ uses a gradient descent method to determine how much a given region (collection of pixels) of an image influences sample classification (e.g., particular MP composition). The result is a contour map showing which parts of the image are the most important, with normalized pixel values ranging from 0 (blue) to 1 (red) indicating importance levels (low, blue; high, red). By superimposing the GradCAM++ contour plots on the experimental optical micrographs, we generated heatmaps of the type shown in Fig. 2C. Inspection of Fig. 2C reveals that the regions of highest importance for classification correlate with the locations of MP clusters. In Fig. 2G, we show representative GradCAM++ heatmaps for sample types from all five MP compositions, where the red regions correlate with MP clusters, confirming that it is the aggregation patterns that enable DeepPolyNet to differentiate between the different MP compositions.

Step 3 of our approach was to determine whether it was possible to identify physical features of MP aggregates used by DeepPolyNet to indicate the composition of the MPs from which the aggregates were formed. To this end, we analyzed the sizes and structures of aggregates or “key clusters” of MPs (Fig. 2, step 3) identified as having high feature importance by GradCAM++ (Fig. 2D) (*50*). Specifically, guided by past studies of colloidal aggregation (*51*, *52*), we used five descriptors of MP aggregates [aggregate (or cluster) size, which is defined as the number of MPs per cluster; perimeter length; circularity; aspect ratio; and fractal dimension (FD)]. We evaluated the relative ability of the descriptors to classify MP aggregates when using machine learning classification algorithms (e.g., random forest and XGBoost) that have been used previously to classify aggregation patterns (fig. S7) (*34*, *53*). In contrast to the CNN used in step 1, these machine learning methods do not process raw image data directly but use preextracted descriptors, such as shape descriptor values, to perform the classification.

The bar plot in Fig. 2E shows the relative ability of all five descriptors mentioned above to predict the composition of the MP mixture from which interfacial aggregates were formed. We found that FD, cluster size (number of MPs in each cluster), and circularity are individually effective descriptors. This result suggests that the MP mixture composition directly affects the morphology of the MP aggregates in ways that are readily identified by quantitative changes in physical shape descriptors. To illustrate this point, we show how the FD distributions change with MP mixture composition in Fig. 2F and Table 1 (results for cluster size and circularity are shown in fig. S6). Inspection of Fig. 2F and Table 1 reveals that aggregates formed at LC interfaces from single-component aqueous MP samples (such as 100% PS or 100% PMMA) are significantly more compact (smaller FD) than those formed from MP mixture (50% mixture composition was used as the reference) samples (higher FD), with a *P* value <0.001 from a *t* test (Table 1). Statistical analysis also shows that the FD distributions for the three different binary mixture compositions (25% PS, 50% PS, and 75% PS) are not significantly different from each other, with a *P* value of 0.078 when comparing 50% mixture types to either 25 or 75% mixture composition types [Table 1 and fig. S8 (for the summary of *t* tests)]. However, the size differences, defined as the number of MP particles per cluster, show significant variation across all five sample compositions (Table 1 and fig. S8). Overall, these findings are consistent with qualitative observations based on visual inspection of the aggregation patterns, as reported earlier here (see the text related to Fig. 1H).

**Table 1.** Morphological analysis of MP aggregates based on shape descriptors, categorized by composition in the aqueous phase at a concentration of 400 mg/liter. The t-test p-value is provided for each sample in comparison to the 50% PS binary mixture, with a sample size of 100 and a significance level of 0.05.

Property	0%PS	25%PS	50%PS	75%PS	100%PS
FD	1.13 ± 0.04	1.20 ± 0.04	1.21 ± 0.04	1.22 ± 0.04	1.11 ± 0.04
Cluster size	23 ± 4	26 ± 5	28 ± 4	30 ± 5	19 ± 5
FD p-value	<0.001	0.078	-	0.078	<0.001
Cluster size p	<0.001	0.002	-	0.002	<0.001

The discussion above addresses the performance of single-shape descriptors in classifying samples that differ in MP composition. However, we found that three shape descriptors, when combined (circularity, cluster size, and FD), substantially improved the sample classification (see fig. S7B). By using a combination of the three descriptors, we found that it was possible to classify the five types of MP samples defined in Table 1 with an accuracy of 97.7%. Overall, this high accuracy of classification based on shape descriptors of the aggregates provides support for our conclusion that DeepPolyNet achieves a high classification accuracy by analyzing the “fingerprints” or characteristic structural features of the aggregates formed by the MPs. However, we also note that the shape descriptor–based classification still falls short of the accuracy provided by DeepPolyNet (99.7% accuracy). The difference in accuracy suggests that the CNN is using additional structural information not captured by the three shape descriptors used in our analysis.

### Do the structures of aggregates of binary mixtures of MPs at LC interfaces evolve predictably with preexposure to UV light?

The question addressed in this section is motivated by the observation that MPs in the environment are subject to aging under natural sunlight. Photolytic degradation of polymeric surfaces is well studied, particularly for PMMA. Prolonged UV irradiation at a wavelength of 254 nm has been reported to change the surface chemistry and topography of PMMA (*54*, *55*) and PS (*56*). Whereas PMMA surfaces undergo substantial surface oxidation within days of UV exposure, PS surface oxidation is comparatively slow and may take 1 to 3 months under similar conditions (*57*–*59*). We hypothesized that changes in the surface chemistry of MPs of PMMA within mixtures of PMMA and PS would be transduced into changes in aggregation behaviors of the MP mixtures at the LC-aqueous interface.

To test this hypothesis, we used a set of UV illumination conditions developed in prior studies (*58*, *60*–*63*) that simulated aging of MP samples under natural sunlight. Specifically, we exposed aqueous saline (0.3 M) dispersions of single-component MPs and equimolar MP mixtures of PMMA and PS to UV irradiation (8-W lamp, wavelength range from 250 to 280 nm with 90% of energy at a peak wavelength of 254 nm; intensity of illumination of samples of 15 W/m^2^) for 3, 5, or 10 days (*61*, *62*). Following UV exposure, the MP dispersions were incubated against LC interfaces for 15 min, and colloidal aggregation patterns were imaged under bright-field optical microscopy.

**Fig. 3.**
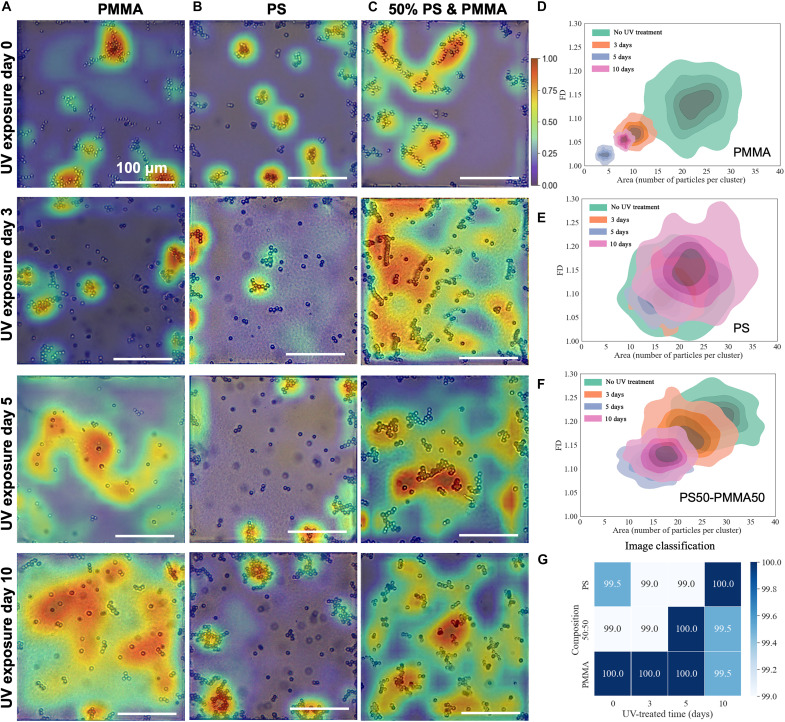
Effect of UV aging on aggregation patterns of single-component and binary mixtures of MPs (PS/PMMA) at the LC-aqueous interface. First, the effect of UV exposure is probed on single-component MPs (PS or PMMA); second, on 1:1 MP binary mixtures. Representative GradCAM++ heatmaps from optical micrographs of aggregation patterns of single-component PMMA [first column (**A**)], PS [second column (**B**)], and an equimolar mixture of PS and PMMA [third column (**C**)] MPs formed at the LC-aqueous interface after exposure to UV irradiation for 0, 3, 5, and 10 days. Aqueous 0.3 M NaCl dispersions (400 mg/liter) of MPs were illuminated by 254-nm-wavelength UV radiation for the above-mentioned exposure times. The color bar from blue to red reflects feature importance from 0 to 1. Scale bars are 100 μm. (**D** to **F**) 2D plots of FD versus area (number of particles per cluster) of aggregates of (D) PMMA, (E) PS, and (F) a 1:1 MP mixture for different durations of UV exposure. (**G**) Confusion matrix showing the overall classification performance for four classes of UV exposure durations and three classes of MP compositions. The numbers within each box in the 4-by-3 matrix represents the accuracy of DeepPolyNet for classifying samples by (i) UV exposure time and (ii) MP composition (mixture versus single-component PS or PMMA) of UV-aged samples.

Figure 3 (A and B) shows optical micrographs of aggregation patterns of UV-aged single-component MPs (PMMA or PS) onto which GradCAM++ heatmaps have been superimposed. Inspection of the regions of the highest feature importance (red) reveals that MP aggregate size decreases with increasing UV exposure for MPs of PMMA (Fig. 3A). After 5 or more days of UV exposure, the PMMA MPs are either singly dispersed or form very small clusters (evident in the day 10 image). In contrast, inspection of Fig. 3B reveals that MPs of PS show no obvious changes in aggregate size before and after UV exposure for up to 10 days. To quantify these observations, we conducted a morphological analysis using DeepPolyNet and shape descriptors (FD and cluster size), as detailed earlier in text describing Fig. 2. For the MPs of PMMA, UV exposure led to a significant reduction in both FD and the number of particles per cluster (Fig. 3D and Table 2). In contrast, PS samples exhibited no significant change in FD or cluster size after UV exposure (Fig. 3E and Table 2).

**Table 2.** Morphological analysis of PS, PMMA, and equimolar PS-PMMA mixture assembly behaviors under UV treatment.

Sample type	Morphological analysis of MP samples under UV treatment					
PMMA	Source	Property	0 day	3 days	5 days	10 days
	Figure 3D	FD	1.13 ± 0.04	1.07 ± 0.01	1.02 ± 0.01	1.05 ± 0.01
		Cluster size	23 ± 4	10 ± 2	4 ± 1	8 ± 1
PS	Source	Property	0 day	3 days	5 days	10 days
	Figure 3E	FD	1.11 ± 0.04	1.10 ± 0.03	1.09 ± 0.02	1.13 ± 0.02
		Cluster size	19 ± 5	18 ± 3	16 ± 3	22 ± 3
Equimolar PS-PMMA	Source	Property	0 day	3 days	5 days	10 days
	Figure 3F	FD	1.21 ± 0.04	1.17 ± 0.03	1.11 ± 0.02	1.13 ± 0.02
		Cluster size	28 ± 4	22 ± 4	15 ± 3	17 ± 3

We also investigated the effect of UV exposure on equimolar mixtures of PS and PMMA (Fig. 3C). After 5 days of UV exposure, the aggregation behavior is characterized by low FD and small cluster size (Fig. 3F). We confirmed that this trend is not caused by a change in the number density of MPs on the interface (fig. S9). It does, however, mirror the behavior of single-component PMMA samples (Fig. 3D) after 5 days of UV exposure (Table 2 and fig. S9). This change in the aggregation behavior of the equimolar PS and PMMA samples is recognized by DeepPolyNet, as shown in the confusion matrix in Fig. 3G, leading to accurate classification of UV exposure time (>99%). DeepPolyNet can also recognize MP composition (mixture versus single-component PS or PMMA) of UV-aged samples with high accuracy (see also Fig. 3G).

**Fig. 4.**
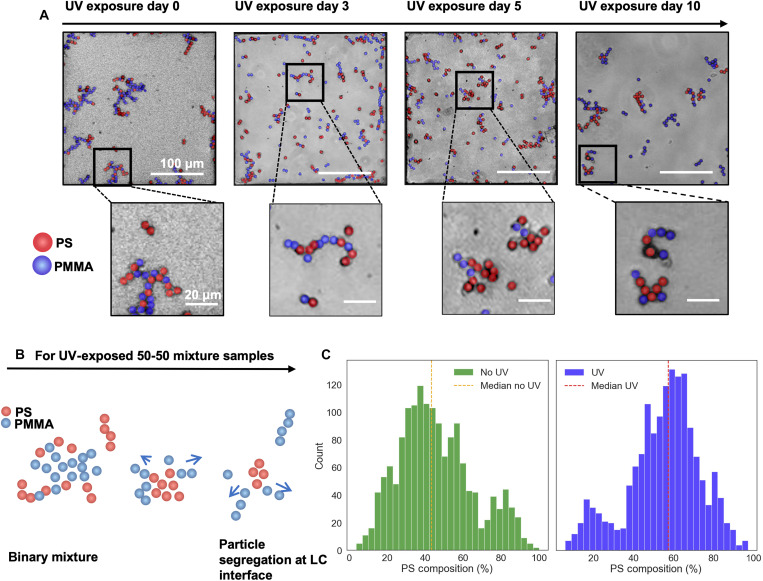
Selective depletion of PMMA MPs from PS-PMMA aggregates by UV illumination. MP assembly analysis of 50% PS-50% PMMA mixture clusters, exposed to different durations of 254-nm-wavelength UV light. (**A**) Shown across the row are false-color micrographs (and corresponding magnified insets) of merged phase contrast and fluorescence images of MP aggregates at the LC-aqueous interface. Scale bars are indicated. (**B**) Schematic illustration of exclusion of PMMA MPs from mixed clusters on UV exposure. (**C**) Plot comparing average PS incorporation in clusters for 50% PS-50% PMMA mixture samples before UV exposure (green, legend: no UV) and after 5 days of UV exposure (blue, legend: UV). The *x* axis shows the distribution of aggregates with PS composition, and *y* axis “Count” refers to the frequency of aggregates with a particular PS composition. All results shown are for MP dispersions (400 mg/liter) in the presence of 0.3 M NaCl.

To gain insight into the physical origin of the UV-induced changes in aggregation status that are recognized by DeepPolyNet (as shown in Fig. 3), we next studied the UV-dependent internal organization of mixed PS and PMMA clusters formed from aqueous dispersions of equimolar mixtures. To characterize the internal organization of the aggregates, we used fluorescently tagged PS MPs (see Materials and Methods and fig. S10). This approach, when combined with fluorescence microscopy, allowed us to identify MPs within aggregates as being PS (fluorescent) or PMMA (not fluorescent). Figure 4A shows a representative set of results, presented using false-colored optical micrographs (see Materials and Methods for details on the MP visualization scheme and fig. S10), with PMMA being indicated as blue and PS shown in red. Inspection of clusters before UV exposure (Fig. 4A, UV exposure day 0) reveals the incorporation of both PS and PMMA MPs. The aggregates are, however, enriched in PMMA, and the PS MPs are typically incorporated as small clusters within or at the periphery of PMMA-rich aggregates (Fig. 4A). With UV exposure, accompanying the reduction in cluster size, as reported above, we observed the composition of the clusters to be depleted of PMMA MPs as compared to PS (Fig. 4, A and B). Specifically, before and after 5 days of UV treatment, the mean % of PS MPs within the aggregates increased from 43 ± 18 to 57 ± 19% (Fig. 4C), a difference that was determined to be statistically significant (*P* value <0.001 for *n* = 1558 clusters). This result hints that UV exposure causes the interactions of PMMA particles to become repulsive, leading to their exclusion from mixed clusters. Below, we return to discuss the physical origin of the UV-induced interactions between the MPs.

### Can we distinguish MPs in the presence of NOM at LC interfaces using DeepPolyNet?

In this section, we report how the presence of NOM influences the aggregation behavior of PS and PMMA MPs at the LC interface and the impact of NOM on classification of MPs. NOM in environmental waters refers to a mixture of inorganic sediments and organic compounds that originate from the natural decomposition of plants and organisms. NOM typically has two components, molecular (inorganic or organic components that dissolve in water) and colloidal (particulate matter that does not dissolve in water) (*9*). Both molecular NOM and colloidal NOM are known to adsorb to MP surfaces and affect MP aggregation. Although it has been shown in past reports that PS nanoparticle aggregation is reduced in the presence of polysaccharides and humic and fulvic acids (*9*, *64*), studies of MP aggregation in the presence of colloidal NOM are sparse (*65*). However, it is established that the presence of NOM complicates existing MP characterization methods (*8*, *9*). Our goal for the experiments described below was to determine to what extent NOM affects a strategy for MP characterization on the basis of aggregation of MPs at LC interfaces.

**Fig. 5.**
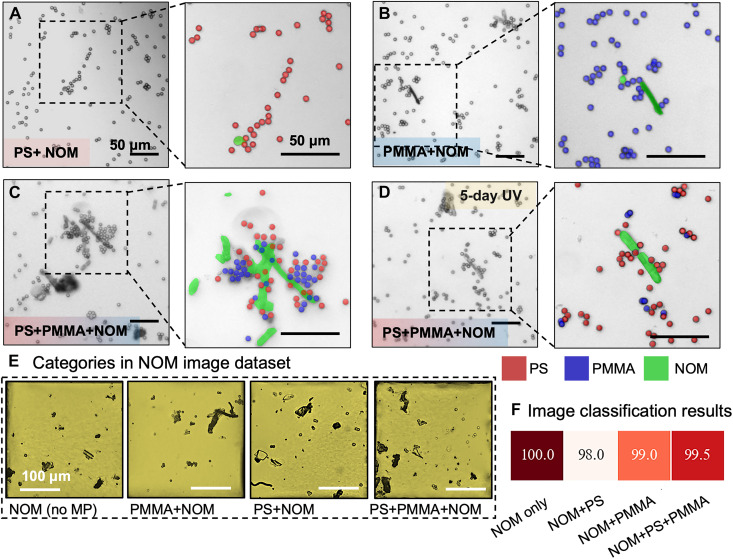
Aggregation of a single component and a binary mixture of PS-PMMA MPs in the presence of NOM. Phase contrast optical micrographs showing the influence of colloidal NOM on organization of a single component (**A** and **B**) and 1:1 mixtures of PS and PMMA MPs before (**C**) and after 5 days of aging under 254-nm UV light (**D**), all in the presence of 0.3 M NaCl. The insets show magnified false-colored images of MP clusters, with NOMs incorporated. The false-colored images are overlays of phase contrast and fluorescence images, where the PS (red) and PMMA (blue) particles were both fluorescently labeled. The NOM debris is colored green. Scale bar are 50 μm. (**E**) Representative bright-field optical micrographs of sample categories in the DeepPolyNet dataset consisting of four sample types (200 images for each sample type and 800 images overall): NOM (no MP), NOM + 100% PMMA, NOM + 100% PS, and NOM + 50% PS-50% PMMA using an MP concentration of 20 mg/liter. Scale bar are 100 μm. (**F**) Results of DeepPolyNet classification.

NOM composition varies across natural environments (e.g., marine water versus lake water containing agricultural runoff) depending on the primary sources of organic matter and the microbiome that initiates a cascade of degradation pathways leading to the formation of the molecular components of NOM. While the literature provides different descriptions of NOM (*66*, *67*), on the basis of a previous report (*68*), we have chosen a primary source that is close to terrestrial peat wetlands or forest runoffs. Specifically, to represent environmental MP samples containing NOM, we used a commercially available potting mix composed of sphagnum peat. In addition to a colloidal component, we extracted water-soluble molecular NOM from peat using a procedure reported previously (*68*). We measured NOM concentrations of 30 to 60 mg/liter for the soluble component and 79 ± 32 mg/liter for the colloidal components. These concentrations lie within ranges reported in environmental samples (see the text accompanying fig. S11 for a discussion of NOM concentrations). Inspection of Fig. 5 (A and B) leads to three key observations regarding the impact of NOM on MP aggregation: (i) We observe that soluble NOM disperses single-component MPs. In particular, in regions far from colloidal NOM, both PS and PMMA MPs showed diminished aggregation compared to NOM-free samples (Figs. 1G and 5, A to C, and fig. S11B). This observation is also supported by additional experiments performed after the removal of larger colloidal particulates of NOM from samples containing MPs (fig. S11A). (ii) We observe the colloidal NOM to directly interact with MPs to change MP aggregation (Fig. 5, A and B, and figs. S11 and S14), including for mixed MPs samples (Fig. 5C and fig. S11). (iii) After UV exposure of samples, we observed the PMMA MPs and colloidal NOM to exhibit a lower level of heteroaggregation. This change in aggregation behavior post–UV illumination of MP mixtures in the presence of NOM mirrors aggregation behaviors observed in the absence of NOM (Fig. 4A), where we see diminished incorporation of PMMA in mixed PMMA and PS clusters. In addition, in figs. S12 and S13, we present the results of experiments performed to characterize the LC orientation around MPs exposed to NOM. These experiments reveal that the LC orientation does not change when PMMA MPs (with or without UV treatment) were exposed to NOM.

To train DeepPolyNet on MP aggregation patterns in the presence of NOM, we used a dataset of bright-field images (compatible with the rest of our DeepPolyNet dataset) of MP aggregates obtained in the presence of NOM (for both single-component MPs and 1:1 MP mixtures; Fig. 5E). We highlight two image classification results shown in Fig. 5F. First, we find that DeepPolyNet can differentiate between MPs (without NOM) and NOM-containing samples (with/without MPs) with 100% classification accuracy (brown box) (fig. S15). Second, in samples containing both MP mixtures and NOM, the algorithm was able to recognize the sample MP compositions (single-component PS, PMMA, or mixture) with high accuracy (Fig. 5F).

While it is expected that DeepPolyNet can distinguish between colloidal NOM and MPs, given the uniformity of MP shape and size in comparison to colloidal NOM, it is interesting that the algorithm can identify the presence of soluble NOM components in the absence of colloidal NOM (Fig. 5A). We have also explored the ability of the algorithm to classify samples with a wider range of NOM sources and colloidal NOM concentrations. The results are detailed in fig. S16. Overall, our results support the proposal that DeepPolyNet is recognizing changes in aggregation patterns of MPs induced by the dissolved NOM components. These results demonstrate that our MP classification methodology can be applied to samples containing NOM, a result that is remarkable because NOM is widely found in environmental samples.

### Evaluation of the ability of DeepPolyNet to address important use cases with MPs

**Fig. 6.**
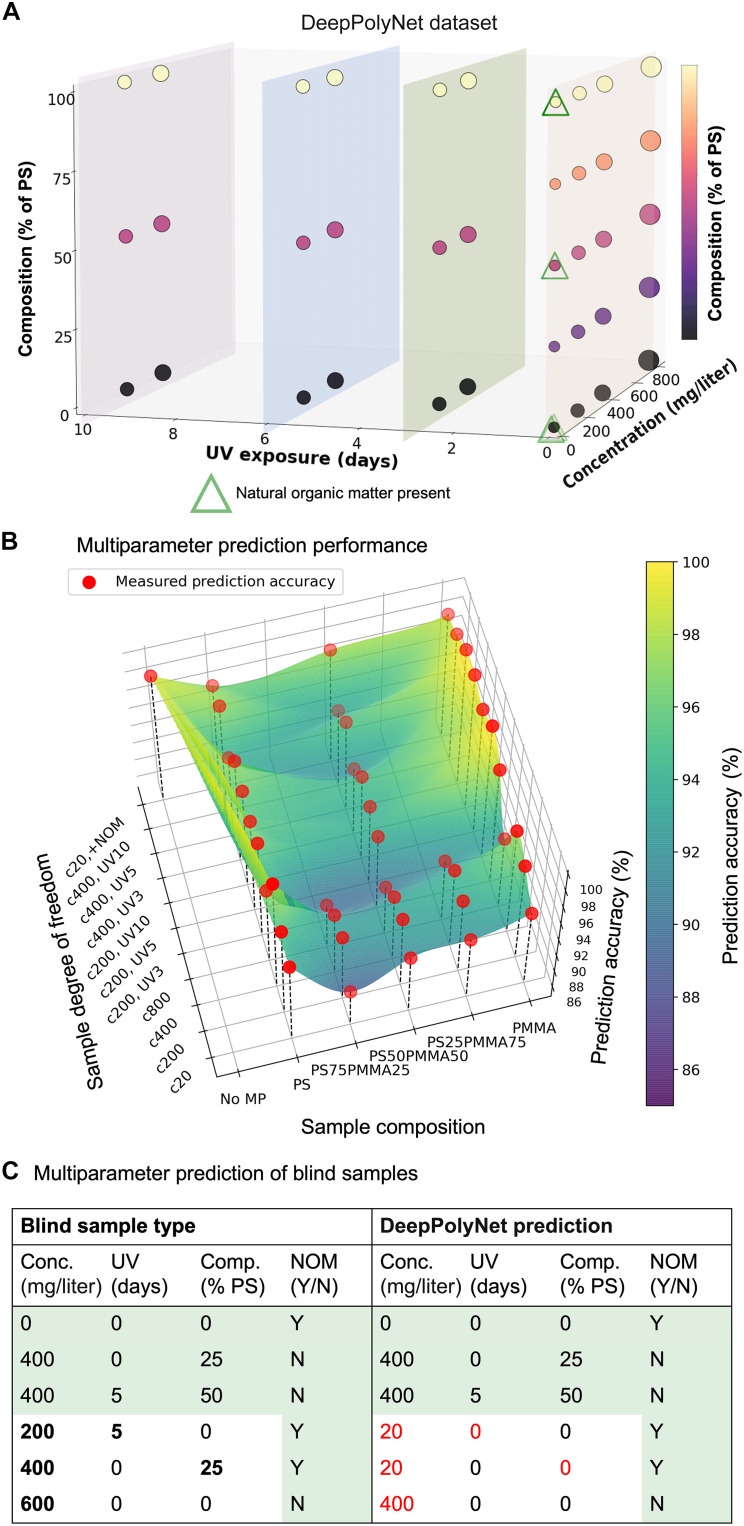
Performance of DeepPolyNet in recovering sample parameters from aggregation pattern analysis. (**A**) Classification framework with four degrees of freedom or parameter categories, (i) composition, (ii) concentration, (iii) UV treatment duration, and (iv) the presence of NOM with six, five, four, and two sample types in respective categories. Each sample type indicated by a data point comprises 200 optical micrographs, with the total dataset comprising 8400 images spread across a total of 42 sample types. These sample types are referred to as “predefined” classes. The samples with NOM are presented as green triangles overlying data points of samples without NOM. The entire dataset was divided into training (70%), validation (20%), and testing (10%) datasets. The color bar indicates the composition of PS (in %) in the PS-PMMA mixture. (**B**) The plot illustrates the multiparameter prediction accuracy for classifying optical micrographs into 1 of the 42 sample types, where accurate predictions require correct identification of sample types within all four categories (use case 2). The color bar from blue to yellow shows prediction accuracies ranging from 85 to 100%. The surface plot represents cubic interpolation of these prediction accuracies for individual sample types, marked by red circles. The prediction accuracy values (fig. S15B) reflect the model’s performance on the testing data. (**C**) Table showing results of DeepPolyNet prediction of blind samples where all four degrees of freedom are unknown, including three samples (bottom three entries in the table) with parameters outside the training sample types. The left column indicates the actual experimental parameters, while the right column indicates the predicted sample types. Incorrectly predicted labels are highlighted in red.

Next, we discuss DeepPolyNet in the context of its ability to address important use cases involving MPs. We have organized the use cases below into two categories. In the first category (use cases 1 and 2), the samples are of a type that was present in the dataset used to train DeepPolyNet (see Fig. 6A). In the second category (use case 3), we use DeepPolyNet to classify a sample of a type that was absent from the training dataset.

Use case 1 represents a scientist who has collected an environmental MP sample and is seeking to determine the MP composition without additional treatment (removal of NOM) of the sample. This user is not interested in knowing whether NOM is present or whether the samples have been aged by UV light. For this user, DeepPolyNet was highly accurate in determining the sample composition across all sample types. Specifically, samples containing single-component MPs of PS or PMMA were recognized with accuracies of 96.9 and 98.2%, respectively, slightly outperforming the recognition of samples containing mixtures of MPs (75% PS: 95.2%; 50% PS: 94.2%; 25% PS: 93.4%) (results are summarized in fig. S15A).

Use case 2 corresponds to a researcher who wants to identify MP composition, concentration, UV exposure, and the presence of NOM in a sample. As noted above, the sample belongs to a sample type present within the dataset used to train DeepPolyNet. For this second use case, DeepPolyNet’s performance is presented in Fig. 6B (confusion matrix in fig. S15B). While achieving good overall recognition of the four sample attributes (yellow-green regions in Fig. 6B), the lowest accuracies were encountered when determining the composition of (i) MP mixtures (blue region of trough) with either high (800 mg/liter) or low (20 mg/liter) MP concentrations (89% for 20 mg/liter and 90% for 800 mg/liter) or (ii) MP mixtures exposed to UV treatment. We hypothesized that the incorrect predictions were caused by grid-to-grid variation in aggregation patterns, arising, for example, from variation in the number of particles on the LC interface or the locations at which the MPs were deposited. To test this hypothesis, we redefined our input samples to be images of four to six grid squares from a single TEM grid incubated under the same unknown aqueous MP sample (instead of using a single grid square). Subsequently, the sample type prediction was chosen as the most frequently predicted set of sample attributes across the collection of individual grid squares within each input sample. In table S3, we show that this approach improves the sample recognition performance and is able to achieve near-100% accuracy for sample categories that show a lower accuracy when using single grids as inputs (in Fig. 6B). In Fig. 6C, we show results of “blind tests,” where MP samples were prepared by two different experimentalists with sample identities withheld from computational collaborators. Correct prediction of all four MP sample parameters across the three blind test sample types (Fig. 6C; top three entries) demonstrates DeepPolyNet’s reliability across independent users.

Use case 3 addresses a scientist who has collected a sample of a type that falls outside the set of sample types used to train the algorithm. In Fig. 6C (bottom three entries), three blind test samples were selected randomly with experimental parameter sets that fall outside the training data. For all three samples (e.g., 25% PS at a 600 mg/liter concentration), the samples were classified as the closest predefined sample type (e.g., 25% PS at 400 mg/liter), indicating the model’s capability to make reasonable predictions for unknown sample types. We note that the algorithm accurately identified the presence of NOM across all blind test samples and classified any samples with NOM as belonging to one of the four predefined sample types containing NOM. However, the algorithm is currently limited to classifying inputs into predefined discrete categories. Future studies could develop an image regression model to predict continuous sample parameters that fall between training categories while also providing uncertainty estimates to better reflect prediction confidence.

## DISCUSSION

**Fig. 7.**
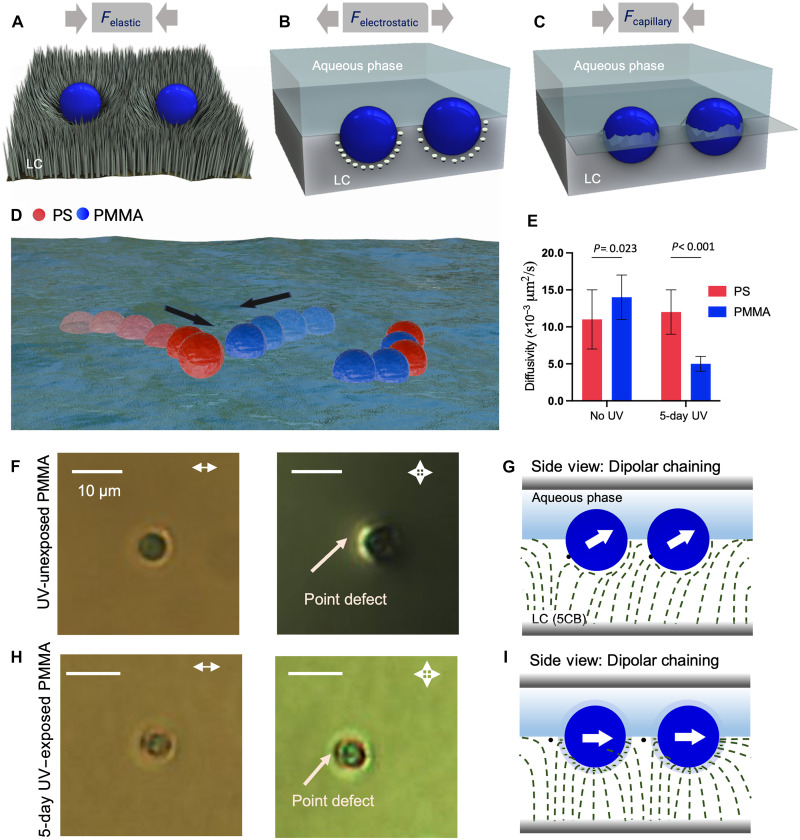
Origin of MP aggregation at the LC-aqueous interface. (**A** to **C**) Schematic illustrations showing three types of interparticle interactions, namely (A) LC elasticity–mediated interaction [more in (F) to (I)], (B) repulsive electrostatic forces as a result of negative surface charge on the particle surface, and (C) attractive capillary forces as a result of contact line pinning. The LC phase and director field is presented in gray for (A) to (C). (**D**) Schematic illustration showing the dynamics of PS and PMMA MPs at aqueous interfaces. Colloidal particles are indicated in blue (PMMA) and red (PS) colors. The black arrow indicates the motion of the particles. (**E**) Bar plot showing the diffusivity of 20 individual particles of PS (red) and PMMA (blue) MPs at the LC-aqueous interface for single-component MP dispersions in the presence of 0.3 M NaCl before UV exposure (no UV) and after 5 days of UV exposure (5-day UV). Error bars are indicated. (**F**) Bright-field (left) and cross-polarized (right) optical micrographs of UV-unexposed single PMMA MPs at the LC-aqueous interface. (**G**) Side-view schematic illustration of topological defect–mediated dipolar chaining of a pair of particles with planar anchoring of LC on the particle surface (5CB on PMMA before UV exposure). (**H**) Bright-field (left) and cross-polarized (right) optical micrographs of 5-day UV–exposed single PMMA MPs at the LC-aqueous interface. (**I**) Side-view schematic illustration of topological defect–mediated dipolar chaining of a pair of particles with perpendicular anchoring of LC on the particle surface (5CB on PMMA after UV exposure for 5 days). LC director field lines are represented by green dashed lines; black dots indicate topological point defects. The experiments were performed in the presence of 0.3 M NaCl. Scale bars are 10 μm.

Motivated by the promising performance of DeepPolyNet for MP sample identification, we end this paper by discussing the role of LCs in mediating the assembly of MPs into distinct aggregation patterns that are recognized by DeepPolyNet. We approach this goal by focusing on understanding changes in MP aggregation patterns measured before and after UV exposure, as documented in our experiments (Fig. 4). Specifically, as discussed in Results, we observed 5 days of UV exposure to (i) disperse PMMA MP clusters in single-component samples and (ii) reduce the incorporation of PMMA MPs into mixed PS-PMMA clusters as well as to generate small clusters. To understand the physical origins of these observations, we have considered three potentially contributing factors as illustrated in Fig. 7 (A to D): first, electrical double-layer interactions between MPs; second, the role of LC elastic strain at the LC-aqueous interface in mediating MP-MP interactions; and third, changes in the mobilities of MPs. Consideration of the third potential factor is motivated by the proposal that the nonequilibrium interfacial aggregate structures formed by MPs are likely influenced by MP mobilities at the LC-aqueous interface. We note that lateral capillary forces as shown in Fig. 7C are important for MPs with rough surfaces (e.g., crystalline polymers such as PE) but less important for amorphous polymers (e.g., PS and PMMA).

### Role of electrical double-layer interactions

**Fig. 8.**
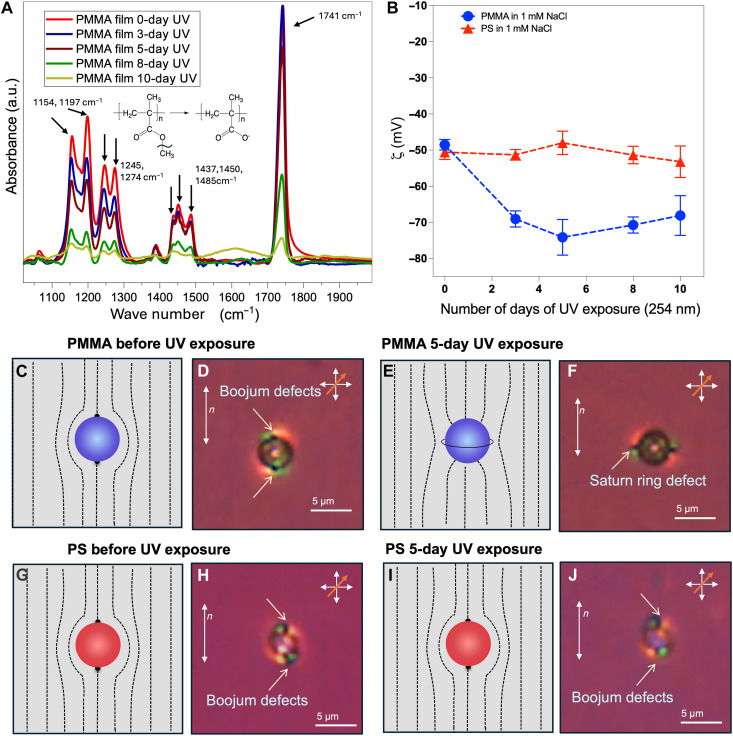
Investigation of LC anchoring on the MP surfaces before and after UV exposure, and surface characterization of MPs. (**A**) Polarization modulation infrared reflection absorption spectra of PMMA films exposed to various durations of 254-nm-wavelength UV light. a.u., arbitrary units. (**B**) Measured values of zeta potential for PMMA and PS MPs in 1 mM aqueous dispersions that were exposed to different durations of 254-nm-wavelength UV radiation. Schematic illustration (**C** and **E**) and optical micrographs (**D** and **F**) of single PMMA MPs before [(C) and (D)] and after 5 days of exposure to 254-nm UV light [(E) and (F)]. Schematic illustration (**G** and **I**) and optical micrographs (**H** and **J**) of single PS MPs before [(G) and (H)] and after 5 days of exposure to 254-nm UV light [(I) and (J)]. The black dashed lines indicate the LC director profile. The optical micrographs indicate optical textures from cross-polarized microscopy using a retardation plate [λ-plate (530 nm)] inserted at 45° with respect to the polarizer in a planar cell with a 27- to 32-μm thickness.

We considered the possibility that the decrease in the extent of aggregation of PMMA MPs following UV illumination (and the decrease in the extent of incorporation of PMMA into mixed PMMA-PS clusters following UV illumination) arises from repulsive charge interactions between MPs caused by UV-driven changes in the surface chemistry of PMMA. In support of this proposal, Fig. 8 (A and B) shows that the surface of PMMA undergoes oxidation during UV exposure under our experimental conditions. Polarization modulation infrared reflection absorption spectroscopy measurements reveal that PMMA surface oxidation occurs through two distinct stages (Fig. 8A). First, photolysis of α-methyl groups (α-CH_3_: reduction of the peaks between 1100 and 1500 cm^−1^) leads to surface enrichment of carboxylate ions (p*K*_a_ = 5; where *K*_a_ is the acid dissociation constant) during the first 5 days of UV exposure. Subsequently, extended UV exposure (10 days) results in the photolysis of the pendant ester group (Fig. 8A; reduction in the C═O peak at 1741 cm^−1^). This progression is also reflected in our zeta potential measurements of MP dispersions in 1 mM aqueous sodium chloride (NaCl) (Fig. 8B). We observed PMMA and PS to exhibit similar zeta potential values before UV exposure (PMMA: −48.5 ± 1.5 mV; PS: −50.6 ± 2 mV).

However, after 5 days of UV exposure, the PMMA MPs exhibited zeta potentials consistent with a buildup of negative surface charge density (−74 ± 5 mV) that was not apparent for PS (−48.5 ± 3 mV). This result provides support to the proposal that the decrease in aggregation of PMMA following UV exposure may reflect repulsive electrical double-layer interactions involving PMMA at the LC interface. We note that quantitative evaluation of electrical double-layer interactions between microparticles at oil-water interfaces is complicated by the low dielectric constant and low ionic strength of the oil phase. In addition, when the oil is an LC, the dielectric properties are dependent on the local ordering of the LC. Prior studies have reported that debye screening lengths in nematic 4-cyano-4′-pentylbiphenyl (5CB) can range from several nanometers to ~10 μm depending on the ionic strength of the LC phase (*69*–*71*). Overall, we conclude that electrical double-layer interactions likely contribute to repulsive MP-MP interactions between UV-illuminated PMMA MPs (Fig. 7B), creating a kinetic barrier to clustering [as observed in past studies of the aggregation of microparticles in bulk aqueous solution, as described by the Derjaguin-Landau-Verwey-Overbeek theory (*72*)]. In addition, however, as described below, changes in the charge status of the MPs also affect the ordering of LCs near the MPs and, thus, LC-mediated elastic interparticle interactions.

### Role of elastic interactions

We observed UV illumination of PMMA MPs to cause a change in the anchoring of 5CB on the particle surface from planar to perpendicular (Fig. 8, C to F). In contrast, 5CB anchoring on PS MPs remained planar independent of whether or not the particles were UV illuminated (Fig. 8, G and H; additional discussion of Fig. 8, C to J, is presented in fig. S17). We interpret the anchoring transition exhibited by 5CB on the PMMA surface to result from charging of the PMMA surface during UV exposure (as described in the preceding section). Briefly, the negative surface charge generates an electrical double layer near the PMMA MP surface, producing a radial electric field, such that the local electric field strength at the PMMA particle surface increases with increasing ionic strength (*71*). This field exerts a torque on the LC in the vicinity of the PMMA surface, causing the LC to align its axis of maximum dielectric constant parallel to the electric field lines. Given that 5CB has a positive dielectric anisotropy (Δε = ε_||_ − ε_⊥_ ~ 10), the LC orients perpendicular to the local surface of the PMMA MP (i.e., homeotropic anchoring) (see fig. S17 for further discussion) (*71*). In a control experiment, when UV-exposed PMMA MPs were dispersed in an LC with a negative dielectric anisotropy [*N*-(4-methoxybenzylidene)-4-butylaniline (MBBA)], we observed that the anchoring of MBBA was tangential to the surface of the UV-treated PMMA particles (fig. S18, A and B). This result is consistent with the predicted influence of a radial electric field on the negative dielectric anisotropy of MBBA. The result provides further support for our interpretation that the observed anchoring transition of 5CB on PMMA is a consequence of a dielectric coupling of the LC orientation with the electric field within the electrical double layer generated at the surface of the UV-exposed PMMA particles. We note that we also measured water contact angles on the PMMA surface exposed to UV light (see fig. S19), and we found the trends in contact angle to mirror the changes in surface chemistry evident from the measurements reported in Fig. 8 (A and B). While it is likely that that there are changes in the hydration of the polymer surface as carboxylate ions are introduced into it by UV irradiation, our results obtained with 5CB (Fig. 8, C to I) and MBBA (fig. S18) support a mechanism of influence on the LC that involves the dielectric anisotropy of the LC and formation of an electrical double layer at the surface of PMMA.

The LC-aqueous interface at which the MPs assemble (e.g., Fig. 1) is part of an LC film with a so-called hybrid configuration, as depicted in Fig. 7 (G and I). When placed at such an LC-aqueous interface, MPs with either planar or homeotropic anchoring are predicted to generate elastic strains in the LC that have dipolar symmetry, resulting in elastic interparticle interactions that have a dipolar character and chaining of MPs (Fig. 7, G and I) (*32*). Consistent with the presence of dipolar elastic interactions between MPs that cause planar anchoring of 5CB, we observed MPs not exposed to UV light to exhibit chaining within 15 min of equilibration (Fig. 1, G and H). In contrast, we observed samples containing UV-exposed PMMA MPs to exhibit limited evidence of chaining after 15 min (Fig. 3, A and C). The absence of chaining after 15 min, however, appears to reflect slowing of the kinetics of clustering, as an extended equilibration time (1 to 2 hours) yielded evidence of dipolar elastic interactions even for UV-exposed samples (fig. S20). The observation that UV-exposed PMMA is kinetically slow to cluster under the influence of attractive elastic dipolar interactions is consistent with the predicted effects of an energetic barrier to aggregation arising from repulsive electrical double layer interactions (see discussion in the section above). In addition, however, it is possible also that the slow kinetics of aggregation observed with UV-treated PMMA samples reflects changes in the mobility of the MPs that accompany changes in the anchoring of LC on the surfaces of the MPs. This perspective is discussed further in the following section.

### Role of MP dynamics at the LC-aqueous interface

Prior numerical simulations of particles in LCs have demonstrated that changes in the anchoring of LCs at the surfaces of the particles (including the strength of anchoring) can change particle mobility (diffusivity) by affecting the apparent viscosity of the local region of LC surrounding the particle (*44*). We explored the possibility that the slow kinetics of aggregation of UV-illuminated PMMA reported in the previous section arises from UV-induced changes in the mobility of the MPs. Specifically, we measured the diffusivity of single MPs of PS or PMMA (*n* = 20 for each type) before and after UV exposure for 5 days. Inspection of Fig. 7E reveals that before UV exposure, PMMA MPs diffuse faster than PS MPs at the LC interface (PS: 0.011 ± 0.004 μm^2^/s; PMMA: 0.014 ± 0.004 μm^2^/s) (*P* = 0.023). After 5 days of UV exposure, the mobility of PMMA MPs is reduced by ~64%, to 0.005 ± 0.001 μm^2^/s, while that of PS is unchanged (PS_UV_: 0.012 ± 0.003 μm^2^/s; *P* = 0.38). This observation suggests, therefore, that the slow kinetics of aggregation observed with UV-treated PMMA likely reflects repulsive electrical double-layer interactions (which create a kinetic barrier to aggregation) and/or changes in the mobility of the UV-treated PMMA particles induced by changes in the surface anchoring of the LC on the surface of the MPs. In particular, for nematic 5CB, there exist three Miesowicz shear viscosities, 129.6 and 37.5 mPa·s for shear flow perpendicular to the zenithal and azimuthal orientations of the LC director, respectively, and 22.9 mPa·s for shear flow along the LC director (*39*). The effective viscosities experienced by particles in bulk 5CB have been found to lie between the two larger Miesowicz viscosities for homeotropic (perpendicular) anchoring of the LC on the particle surface or close to the smallest Miesowicz viscosity for parallel anchoring of 5CB on the particle surface. By using the Stokes-Einstein equation, we calculate the apparent viscosities experienced by the PMMA MPs to change by a factor of 2.2 (4.8 to 10.5 mPa·s) with the change in anchoring of 5CB from parallel to perpendicular on the PMMA surface, a change that is similar to that reported in prior studies (1.2 to 2.4 times) for colloids (micro- and nanoparticles) in bulk 5CB (*43*, *48*). We note, however, that the apparent viscosities reported by the MPs at the LC-aqueous interface are smaller than the Miesowicz viscosities, consistent with partial immersion of the particles in the LC phase (the viscosity of bulk water is 0.87 mPa·s at 26°C). Overall, the experimentally observed MP aggregation patterns that enable characterization of MPs by DeepPolyNet can be understood within a nonequilibrium framework that considers the effects of charge interactions and local LC ordering on both MP mobility and LC-mediated elastic interparticle forces.

### Key insights and future directions

In summary, we demonstrate how aggregation patterns exhibited by complex MP samples at LC interfaces can be used to determine key characteristics of the samples relevant to environmental sampling, including the MP concentration, composition, UV exposure time, and the presence of NOM. The approach is based on using computer vision–based methods to recognize spatial features of the aggregation patterns exhibited by the MPs at aqueous-LC interfaces. A noteworthy accomplishment of our investigation is that we are able to provide insights into the physical origins of the unique aggregation patterns that allow the MP samples to be characterized by using computer vision methods. Specifically, we find that UV exposure of MPs of PMMA leads to charging of the PMMA surface, which, in turn, triggers a transition from tangential to homeotropic anchoring of 5CB and changes in the mobility of the MPs at the LC-interface. We conclude that the MP aggregation patterns are recognized by computer vision tools because they uniquely reflect complex MP dynamics encoded by LC ordering around the MPs and interactions arising from an interplay of LC elasticity and electrical double-layer interactions through the LC.

The advances reported in this study also enable emerging directions of research at the intersection of colloid science and environmental monitoring. First, key insight from our current study is that the duration of UV illumination can tailor interparticle interactions at LC interfaces, allowing control over MP composition in binary MP clusters (e.g., PMMA incorporation in PS/PMMA clusters) and morphological parameters such as aggregate size distribution in single-component MP clusters (e.g., PMMA MP clusters). This finding suggests a future direction of inquiry where UV illumination is leveraged as a tool to guide colloidal structure formation at LC interfaces and enable programmable assembly of metastable structures with useful optical and mechanical properties (*73*, *74*).

Second, while we have chosen 5CB as the LC for our experiments, a direction of future investigation could be to explore different LC chemistries as an approach to tune interactions between the LCs and MPs. Past studies have demonstrated, for example, that the choice of LC can influence hydrogen bonding with surfaces (*75*) or the orientational response of LCs to interfacial charge (via changes in the dielectric properties of the LC) (*71*, *76*). The refractive indices of the LCs could also be selected to tune van der Waals interactions between LCs and MPs (*77*).

Third, on increasing MP concentration beyond 800 mg/liter, we observed kinetically arrested percolating networks of MPs at the LC interface (Fig. 1G). Future studies can potentially expand the artificial intelligence toolkit beyond classification of MPs to predict material (e.g., rheological) property evolution of colloids confined to interfaces, including enhancing our understanding of complex phase behavior (*76*) or pathways of colloidal assembly at interfaces leading to gelation (*52*, *77*).

Fourth, we observed that large NOM particles appear to serve as sites at which MPs accumulate, whereas the soluble components of NOM cause MPs to disperse. This finding suggests that a potentially productive direction of future investigation could be to explore in more detail how the soluble and colloidal NOM components combine to influence the aggregation behavior of MPs.

Fifth, we demonstrate that machine learning can be used to simultaneously identify the impact of four key variables on aggregation patterns exhibited by MPs at LC interfaces. These results suggest that productive directions for future investigation could include consideration of diverse MP shapes, including fibers, fragments, and films, or treatment of other irregular surface features of MPs that reflect the diversity of environmental samples. Expanding our approach to the analysis of more complex mixtures, such as ternary systems of PE, PP and PET, or MP mixtures containing mineral particulates, will advance the methodology reported here toward the treatment of environmental MP samples. Last, understanding how the surface chemistry of MPs changes in the presence of microbial films or chemical adsorbates and subsequently affects the interaction between MPs at LC interfaces is a key fundamental challenge critical for capturing a more complete picture of aggregation behavior of environmental MPs at LC interfaces. Ultimately, we envision a handheld field-deployable sensing device that integrates microfluidic circuits for sample handling and permits the capture and analysis of MP aggregation patterns generated at LC interfaces with a smartphone camera and app.

## MATERIALS AND METHODS

### Materials

Detailed information regarding the sources of materials in this study can be found in the Supplementary Materials.

### Overall experimental approach

Figure 1A shows a schematic illustration of our experimental setup (see also fig. S1 for additional details), which comprises a millifluidic channel used to deliver aqueous dispersions of MPs to 20-μm-thick films of the nematic LC 5CB (chemical structure in Fig. 1C). The LC films are hosted within metal (Cu) TEM grids (Fig. 1A), which are supported on silanized glass substrates. Each square of the grids has a lateral dimension of 284 μm by 284 μm, which is large compared to the size of the MP particles used in our experiments (diameters of 5 μm). The supporting glass substrates are coated with octyltrichlorosilane to induce a perpendicular alignment of the LC at the LC-glass interface. Because PS and PMMA particles are denser than water (1.05 and 1.18 g/cm^3^, respectively), they sediment downward onto the LC interface. An electrostatic barrier to the adsorption of MPs at the LC-aqueous interface is screened by the addition of 0.3 M NaCl (*34*). Upon contact of the aqueous dispersion of MPs with the flat LC interface, we observed spontaneous adsorption of MPs, followed by self-assembly into 2D aggregation patterns (schematic illustration in Fig. 1, E and F). We used bright-field optical microscopy (representative examples of optical micrographs in Fig. 1, G and H) to image the self-assembly patterns after 15 min of sample incubation, a time that was selected to ensure that the MPs had time to adsorb and organize laterally. Detailed information regarding the methods in this study can be found in the Supplementary Materials.

### Aqueous extraction of NOM from peat

The water-soluble organic matter was extracted by adding 1 g of potting mix to 500 ml of Milli-Q water in a 1000-ml glass bottle. The obtained mixture was left under shaking at 120 rpm for 24 hours. It is to underline that the aim of such a procedure was not to extract the whole humic substances from the peat soil but only to obtain the water-soluble fraction. The obtained mixture was then spun down at 4000 rpm for 10 min. This was repeated for three cycles after which the supernatant was collected. A 0.3 M NaCl solution was prepared using the extracted supernatant dispersion, and the MP dispersions were prepared using this stock solution. We note that in addition to molecular water–labile NOM, this procedure also introduced colloidal-sized peat debris in the samples that was visible under bright-field optical microscopy.

We describe two sequential experiments using the NOM dispersion, which are also presented in Fig. 5. First, we performed experiments on three different sample types involving incubation of NOM dispersion (concentration, 2 g/liter; 100 times more than the MP weight) with MPs: (i) PS, (ii) PMMA, and (iii) a 50 wt % PS-PMMA mixture using a total MP concentration of 20 mg/liter in each sample, as shown in Fig. 5 (A to C). We used NOM in abundance to mimic environmental conditions, where the concentration of MPs is often much lower than that of NOM. In addition, a low MP concentration also made it challenging for our classification algorithm to identify MPs in NOM dispersions. Second, we wanted to test whether the impact of UV exposure on MP aggregation was substantially altered in the presence of NOM. For this, we exposed the NOM dispersion in the presence of 50 wt % mixture MP samples to UV light for 5 days (following the procedure reported earlier) followed by incubation at the LC interface (Fig. 5D). We used a combination of phase contrast and fluorescence-based imaging using fluorescently labeled PS and PMMA MPs to observe the aggregation patterns of MPs in the presence of NOM. Fluorescence labeling also let us differentiate and observe the distribution of PS and PMMA MPs around colloidal NOM (similar to that in Fig. 4A; images in Fig. 5).

### Safety statement

No unexpected or unusually high safety hazards were encountered.

## Supplementary Materials

### This PDF file includes:

Figs. S1 to S20 Tables S1 to S3 Supplementary Methods and Materials References
