# Supplementary material for: Liquid crystal–driven interfacial ordering of colloidal microplastics: Advancing microplastic characterization below the macroscale
Source: Sci Adv. 2025 Dec 12;11(50):eady1167. doi: 10.1126/sciadv.ady1167 (PMC12700194; doi:10.1126/sciadv.ady1167)
Supplement: Supplementary file 1 — Figs. S1 to S20 Tables S1 to S3 Supplementary Methods and Materials References [file sciadv.ady1167_sm.pdf]

Science Advances
AAAS

Supplementary Materials for

Liquid crystal–driven interfacial ordering of colloidal microplastics: Advancing microplastic characterization below the macroscale

Fiona Mukherjee *et al.*

Corresponding author: Nicholas L. Abbott, nabbott@cornell.edu; Fengqi You, fengqi.you@cornell.edu

Sci. Adv. **11**, eady1167 (2025)
DOI: 10.1126/sciadv.ady1167

This PDF file includes:

Figs. S1 to S20
Tables S1 to S3
Supplementary Methods and Materials
References

SI. 1. Experimental setup for microplastic (MP) adsorption and aggregation on liquid crystal (LC)-aqueous interface

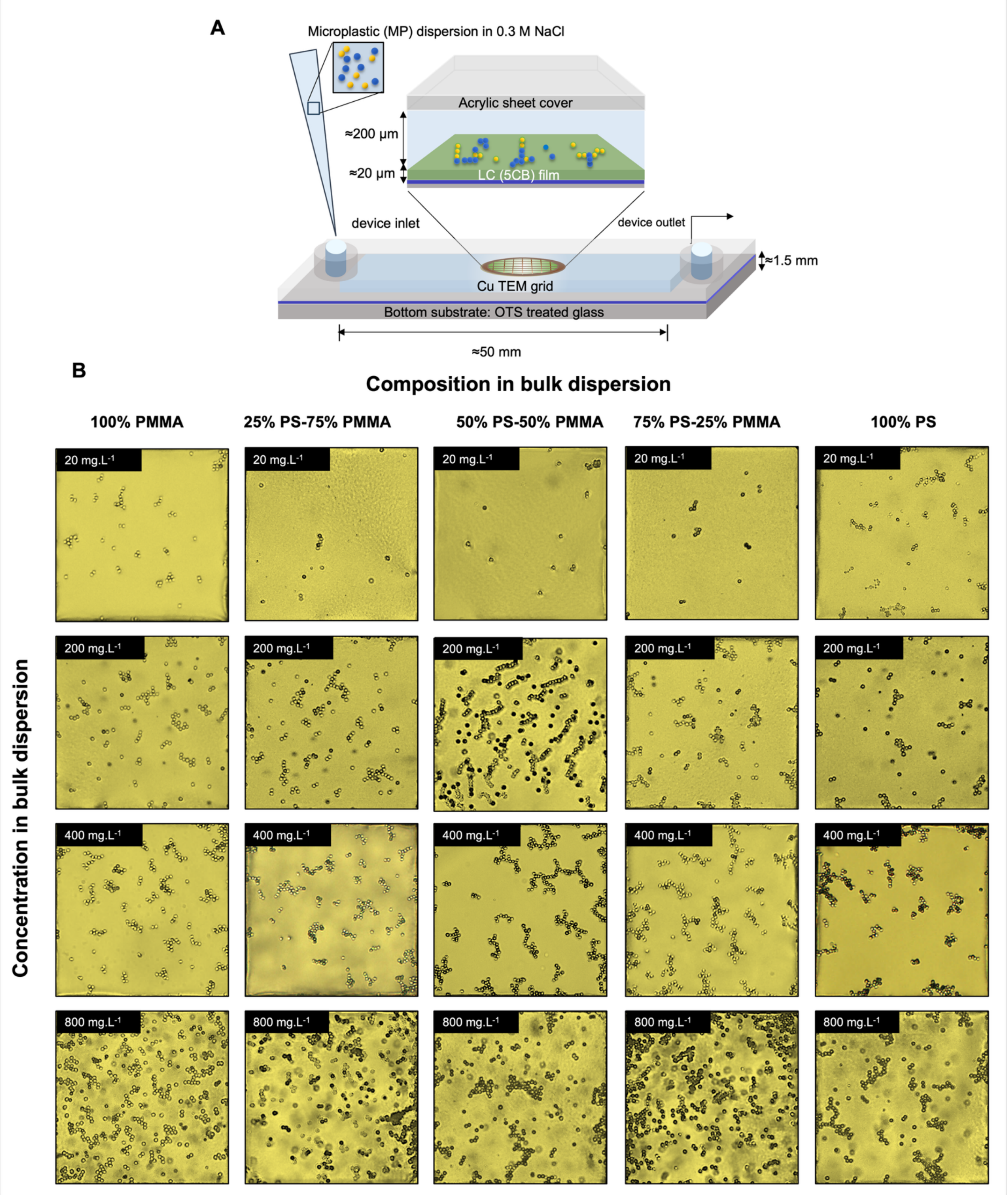

Figure SI. 1: (A) Schematic illustration of a millifluidic channel used to obtain images of microplastics (MPs) adsorbed to LC-aqueous interfaces. (B) 5x4 matrix, showing the brightfield optical microscopy images of LC-aqueous interfaces with different concentration and composition of binary mixture of MPs, polystyrene (PS) and poly (methyl methacrylate) (PMMA). In particular, we vary the concentration of MPs in the aqueous dispersion between 20 mg/L, 200 mg/L, 400 mg/L, and 800 mg/L (along columns) for samples with 5 different compositions (across rows). Each grid square is 284 x 284 μm^2. Five of the twenty images in panel B are duplicates of four images shown in panels G and H of Figure 1 (main text). The four images from Figure 1 (main text) are included in panel B to clearly communicate the effect of concentration and composition on the aggregation of the MPs observed in experiments.

SI. 2. Distribution of MP density in each TEM grid square for different concentrations

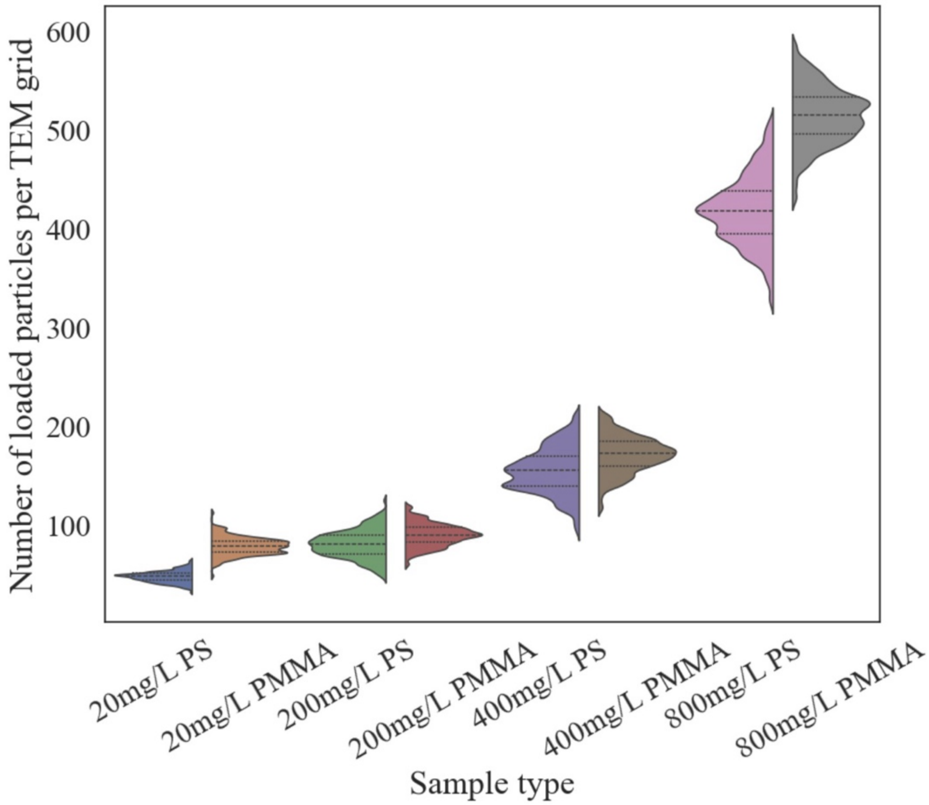

Figure SI. 2: The plot shows the distribution of number of MPs per TEM grid square corresponding to different concentrations of MP aqueous dispersion for both single component PS and PMMA MP samples. Concentrations range from 20 mg/L to 800 mg/L.

SI. 3. Impact of high MP concentration on MP aggregation behavior at LC-aqueous interface

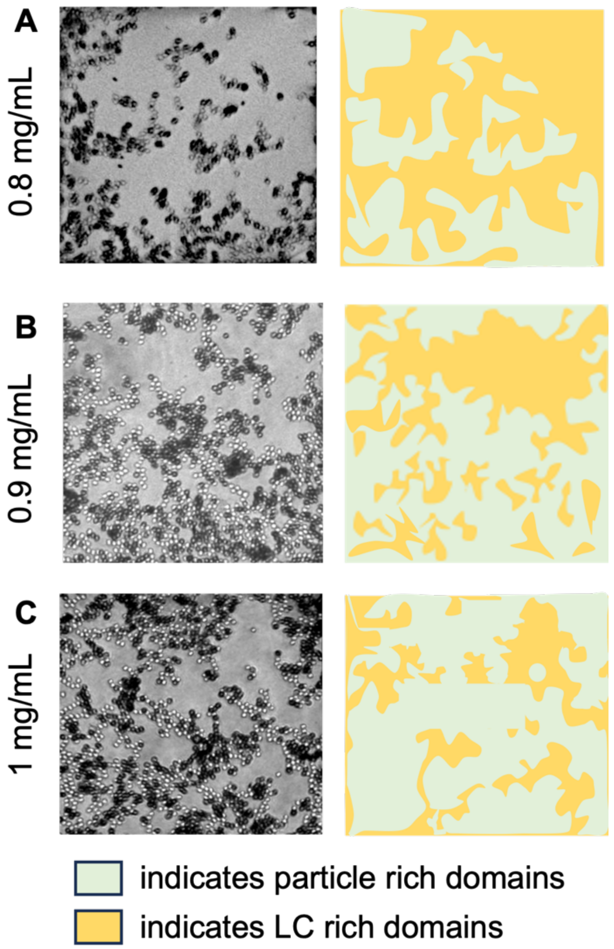

Figure SI. 3: Brightfield optical micrographs of 1:1 PS-PMMA MP mixtures in the presence of 0.3 M NaCl at (A) 0.8 mg/mL, (B) 0.9 mg/mL and (C) 1 mg/mL MP concentration in the aqueous dispersion. The cartoons in the right column visually represent the particle rich and LC rich domains at the LC-aqueous interface. Each grid square is 284 x 284 μm^2. Figure SI. 3C (left image) is duplicated in Figure 1 panel G (main text) and is included here to enable comparison of aggregation patterns as a function of MP concentration.

SI. 4. CNN architecture and GradCAM++ analysis

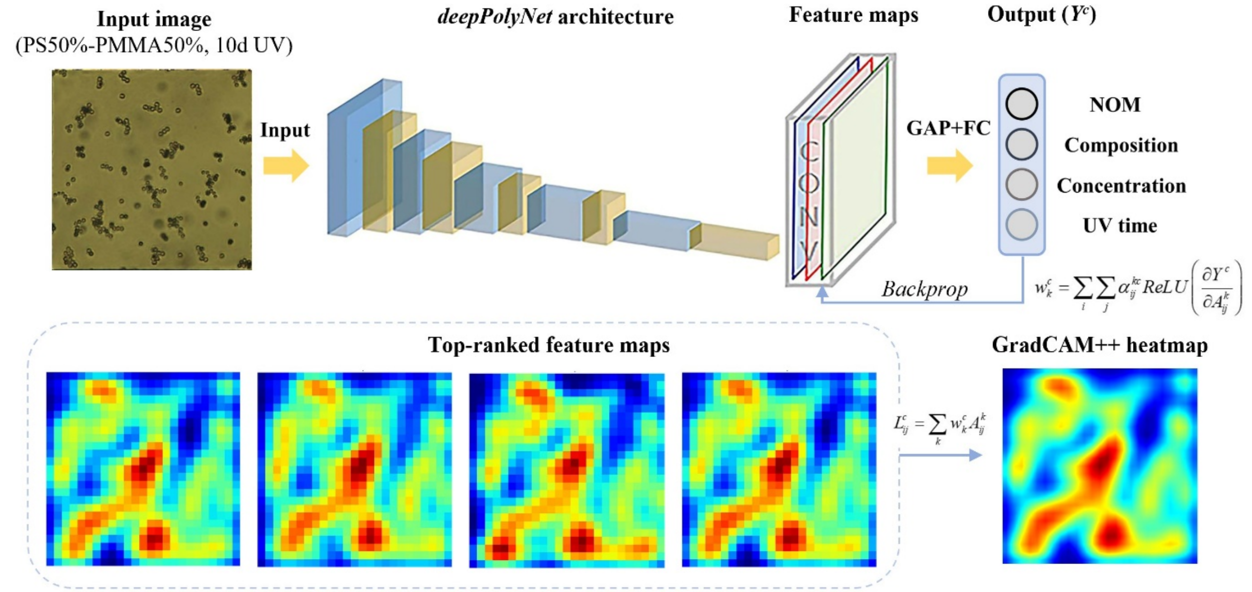

Figure SI. 4: Scheme of *DeepPolyNet* and GradCAM++ analysis. Note that the first image in the top row of Figure SI. 4 is an optical micrograph of 50% PS and 50% PMMA MP aggregates (raw data). The last image in Figure 3C (main text) is a heatmap calculated from this optical micrograph.

The input image is passed through a CNN model, *DeepPolyNet*, for image recognition, followed by a feature importance analysis algorithm, GradCAM++ (*50*), which enables interpretation of the basis of classification by *DeepPolyNet*. *DeepPolyNet*'s architecture is inspired by the previously reported CNN, VGG16 (*78*) which was further tailored to handle the specific challenges of our dataset. *DeepPolyNet* consists of 16 convolutional layers, which are organized into blocks, each block containing multiple convolutional layers followed by a rectified linear unit (ReLU) activation function. The convolutional layers are designed to extract increasingly complex features from the input images, starting with simple edge detectors in the initial layers and progressing to more abstract patterns, such as texture and shape, in the deeper layers. To maintain spatial resolution and allow the model to capture fine details, we use smaller 3x3 convolutional filters throughout the network. This approach enables the network to learn more precise features by focusing on local regions of the image while maintaining computational efficiency. Additionally, the use of max-pooling layers after each block reduces the dimensionality of the feature maps, ensuring that the most salient features are retained while reducing the risk of overfitting. To further mitigate overfitting, we replaced the traditional fully connected layers with a global average pooling (GAP) layer. Unlike fully connected layers that flatten the feature maps into a long vector, which can lead to overfitting in deep networks like VGG16, the GAP layer computes the average of each feature map, resulting in a more compact and generalizable representation (*79*). This design choice not only reduces the number of parameters but also preserves spatial information, which is crucial for accurately identifying aggregation patterns.

Finally, the output of the global average pooling layer is fed into a softmax layer that classifies the images into different aggregation pattern categories. The *DeepPolyNet* models are trained on two different styles in this work: (1) Single label classification, which gives one-dimensional output to recognize one degree of freedom in sample types (e.g., the composition of input image). (2) Multi-label classification for four degrees of freedom recognition. The detailed architecture of *DeepPolyNet*, including the number of filters,

kernel sizes, and the arrangement of layers, is illustrated in Supplementary Table S1. This architecture was carefully designed and fine-tuned through extensive experimentation to achieve optimal performance on our specific dataset, ensuring robust and accurate recognition of MP aggregation patterns.

Table S1. The architecture of *DeepPolyNet*

Layer Type	Filter Size / Stride	Number of Filters	Output Dimensions
Input Layer	-	-	400 × 400 × 3
Conv Layer 1	3 × 3 / 1	64	400 × 400 × 64
Conv Layer 2	3 × 3 / 1	64	400 × 400 × 64
Max Pooling 1	2 × 2 / 2	-	200 × 200 × 64
Conv Layer 3	3 × 3 / 1	128	200 × 200 × 128
Conv Layer 4	3 × 3 / 1	128	200 × 200 × 128
Max Pooling 2	2 × 2 / 2	-	100 × 100 × 128
Conv Layer 5	3 × 3 / 1	256	100 × 100 × 256
Conv Layer 6	3 × 3 / 1	256	100 × 100 × 256
Conv Layer 7	3 × 3 / 1	256	100 × 100 × 256
Max Pooling 3	2 × 2 / 2	-	50 × 50 × 256
Conv Layer 8	3 × 3 / 1	512	50 × 50 × 512
Conv Layer 9	3 × 3 / 1	512	50 × 50 × 512
Conv Layer 10	3 × 3 / 1	512	50 × 50 × 512
Max Pooling 4	2 × 2 / 2	-	25 × 25 × 512
Conv Layer 11	3 × 3 / 1	512	25 × 25 × 512
Conv Layer 12	3 × 3 / 1	512	25 × 25 × 512
Conv Layer 13	3 × 3 / 1	512	25 × 25 × 512
Max Pooling 5	2 × 2 / 2	-	12 × 12 × 512
Global Average Pooling (GAP)	-	-	1 × 1 × 512
Fully Connected 1	-	100	1 × 1 × 100
Fully Connected 2	-	256	1 × 1 × 256
Fully Connected 3 (Output)	-	Number of Classes	1 × 1 × (Classes)
Softmax Layer	-	-	1 × 1 × (Classes)

In the second step of analysis (Figure 2C, Step 2), our goal was to identify the key features used by *DeepPolyNet* for image-based classification. We employed an advanced feature importance analysis algorithm, GradCAM++, to visualize and identify the key visual features within each sample type (e.g., composition) that contribute to accurate classification, as demonstrated in the bottom row of Supplementary Figure SI. 4.

In our CNN architecture, GradCAM++ operates by calculating the gradients of the target class (correctly recognized output sample type) with respect to the feature maps in the final convolutional layer. These gradients are then used to determine the importance of different regions within the feature maps. GradCAM++ further refines this process by considering higher-order derivatives, which enhances the accuracy of localization, particularly when multiple objects or instances are present in the image. This

visualization is represented as contour plots, with normalized pixel values ranging from 0 to 1, indicating the level of importance for image classification from low (blue) to high (red).

SI. 5. Evaluation of *DeepPolyNet*'s image classification performance benchmarked against other CNN models

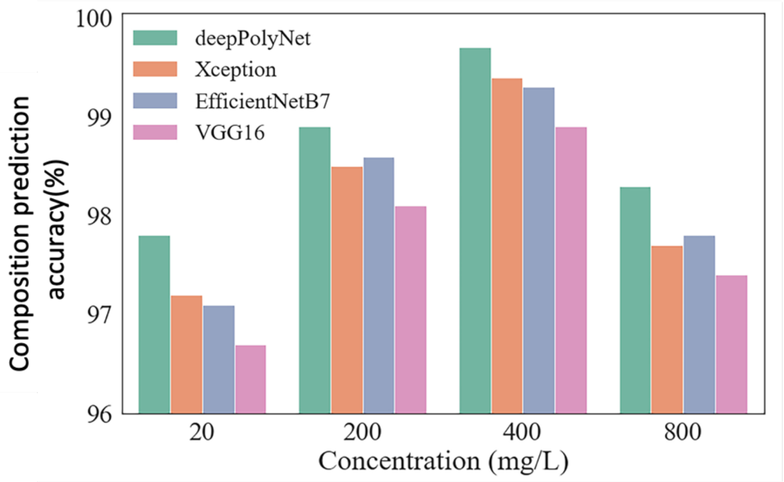

Figure SI. 5: This plot reports the average MP composition recognition performance (y-axis) for sample types with different MP concentrations (x-axis), using different CNN models represented in different colors. In particular, each concentration type comprises images of five different compositions of MP mixtures and each bar plot represents the accuracy of sample composition recognition, averaged over all 5 composition types.

The performance of *DeepPolyNet* is benchmarked against several CNN models, including Xception, EfficientNetB7, and VGG16. We also introduce the Vision transformer model (ViT-b16) for comparison. We trained the CNN and ViT models for both single-parameter (e.g. MP mixture composition, concentration, etc.,) and multi-parameter classification. In the single-parameter case, we developed four separate models, each trained to classify one specific type of parameter: concentration, composition, duration of UV exposure, or presence of NOM. In the multi-parameter case, a single model was trained to predict all four sample parameters simultaneously for each image. The results are presented in Figure SI. 5 and Table S2. *DeepPolyNet* demonstrates improved classification accuracy as compared to the other three selected CNN and ViT models. We attribute this to several factors: first, VGG16's relatively simple and uniform architecture, consisting of sequential small convolutional filters (3×3), is well-suited for smaller datasets and avoids overfitting compared to deeper or more complex networks. Second, models like ViT typically require larger datasets to fully leverage their self-attention mechanisms and often underperform when data is limited. By contrast, VGG16's convolutional design effectively captures local spatial features even with limited training samples, making it a better fit for our dataset of five classes with 200 images per class.

SI. 6. Comparison of *DeepPolyNet*'s sample composition recognition performance with/without cluster selection

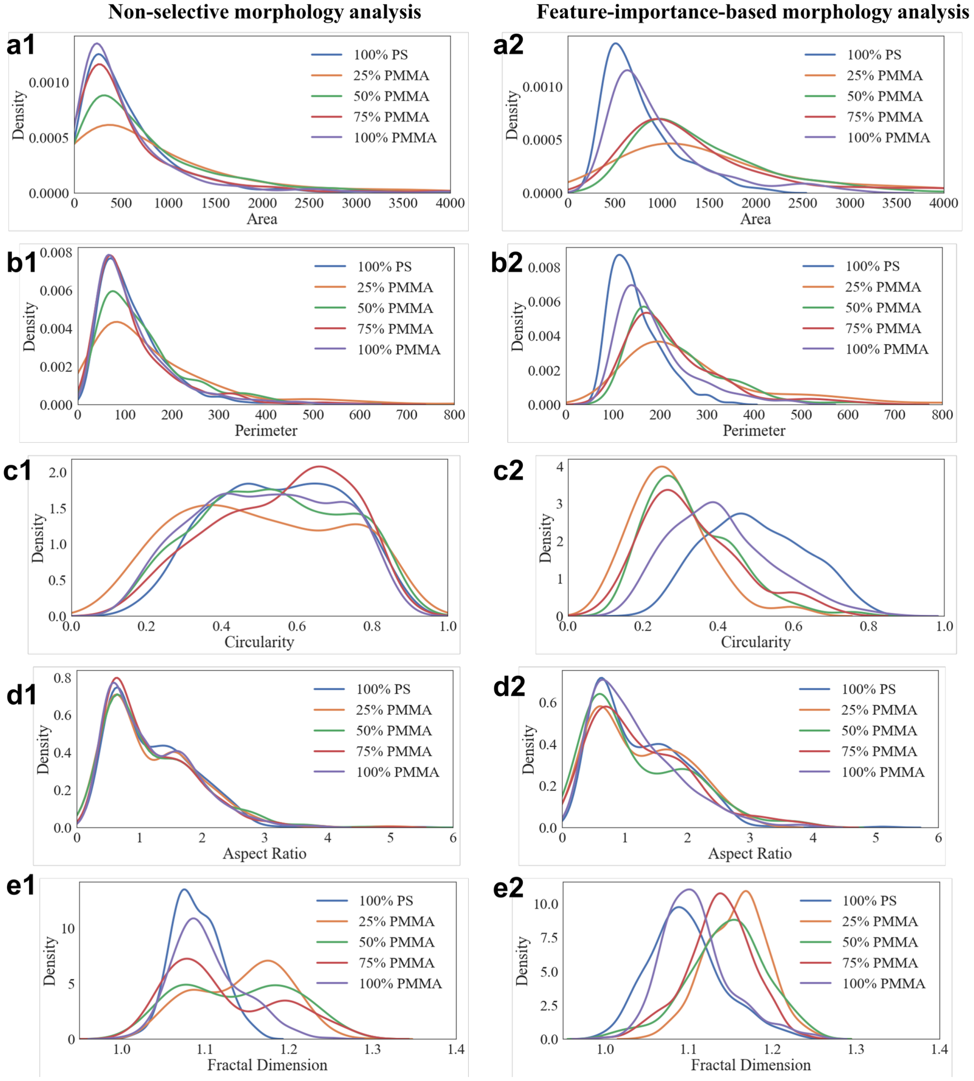

Figure SI. 6: Categorical distributions with/without cluster selection. Left column shows plots of distribution of selected shape descriptors values for all clusters (a1: Area, b1: Perimeter, c1: circularity, d1: Aspect Ratio, e1: Fractal Dimension (FD)) for sample types with different MP mixture composition. Right column shows distribution of selected shape descriptors for "key clusters" only for all 5 classes of MP mixture composition. The plot suggests that selecting "key clusters" leads to better differentiation of aggregate types between samples with different MP compositions. All plots have the same legend.

Table S2. Benchmarking image recognition accuracy of CNN models for single-parameter and multi-parameter classification on the entire MP dataset of 8400 images.

	DeepPolyNet	Xception	EfficientNetB7	VGG16	ViT-b16
Composition	0.994	0.984	0.984	0.978	0.956
Concentration	0.991	0.984	0.984	0.979	0.972
UV-treatment	0.988	0.980	0.978	0.978	0.982
NOM addition	1.000	1.000	0.994	1.000	0.998
Multi-parameter	0.949	0.925	0.922	0.934	0.938

The particle aggregation process involves multiple stochastic factors, leading to the formation of clusters with varying sizes and shapes. This heterogeneity in aggregation makes it challenging to distil out characteristic assembly patterns if all the clusters within a grid square are analysed for each sample type. As shown in Supplementary Figure SI. 6a1-e1, small clusters are predominant, but they are not representative of each sample type. Consequently, the quantitative results of measuring clusters using physical shape descriptors show significant overlap across different MP composition types, making it difficult to discern meaningful differences. Therefore, identifying representative clusters for each class before quantifying their structures is crucial for meaningful comparison between different sample types.

As outlined in step 3 of Figure 2, we developed a cluster selection method that screens for feature importance. Only clusters with high feature importance are further analyzed in terms of their cluster shape. This approach enhances distinctions between MP sample types, as seen in Supplementary Figure SI. 6a2-e2, where the differences in sample type distributions become more pronounced upon cluster selection. To assess the impact of cluster selection, we compared the average intersection over union (IoU) values for five shape descriptors or morphological features (area, perimeter, circularity, aspect ratio, and fractal dimension) before and after selection. Before selection, the IoU values for these features were 0.86, 0.88, 0.91, 0.95, and 0.64, respectively. After cluster selection, the values decreased to 0.71, 0.69, 0.64, 0.81, and 0.50. This reduction in IoU indicates that the overlap in morphological distributions between groups is significantly reduced after cluster selection, allowing for clearer distinctions between experimental conditions.

SI. 7. Classification performance of *DeepPolyNet* when combinations of shape descriptors are considered.

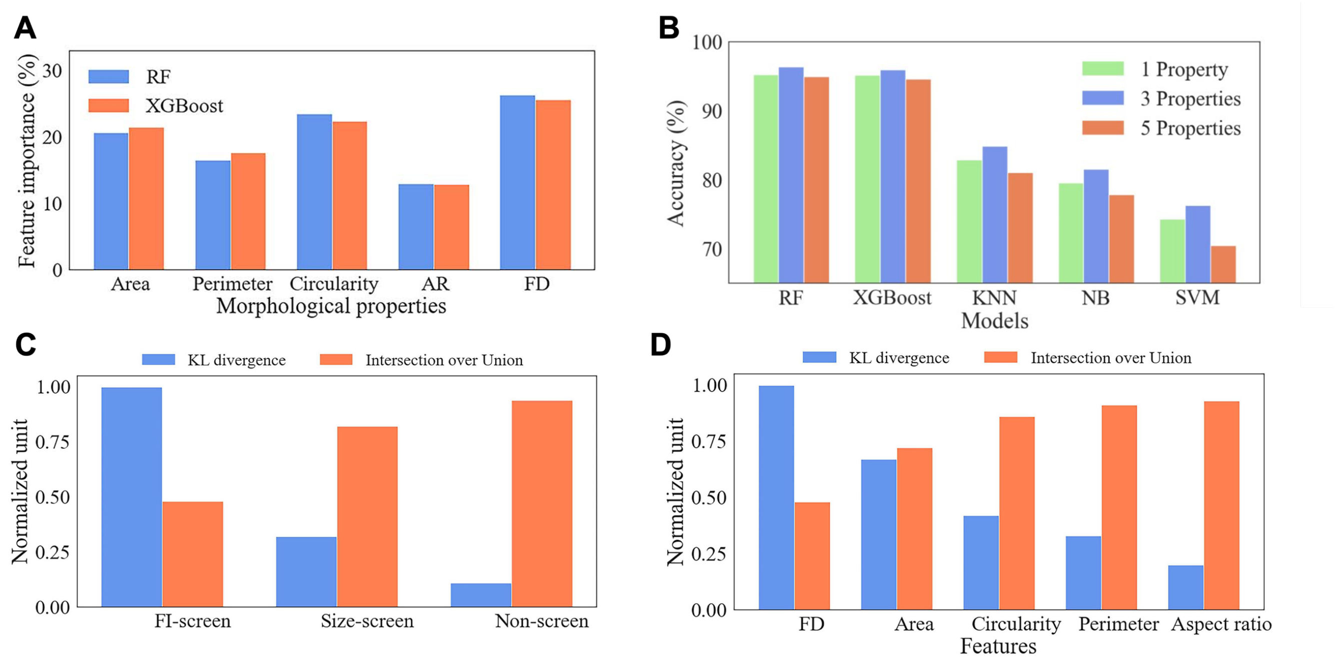

Figure SI. 7: Morphological analysis of MP aggregates using machine learning classifiers. (A) Feature importance analysis of five morphological properties using random forest classifier and XGBoost classifier. (B) Machine learning classification performance using single and combinations of 3 and 5 shape descriptors. (C) The normalized KL divergence and Intersection over Union (IoU) for FD distributions between single component PMMA and PS using different cluster selection strategies: feature importance (FI), aggregate size (Size-screen) and without cluster specification (Non-screen). (D) The normalized KL divergence and Intersection over Union (IoU) for distributions of different morphological descriptors between single-component PMMA and PS clusters using feature importance (FI) based screening.

SI. 8. Comparison between sample types comprised of different MP compositions using physical shape descriptors: fractal dimension (FD) and cluster size

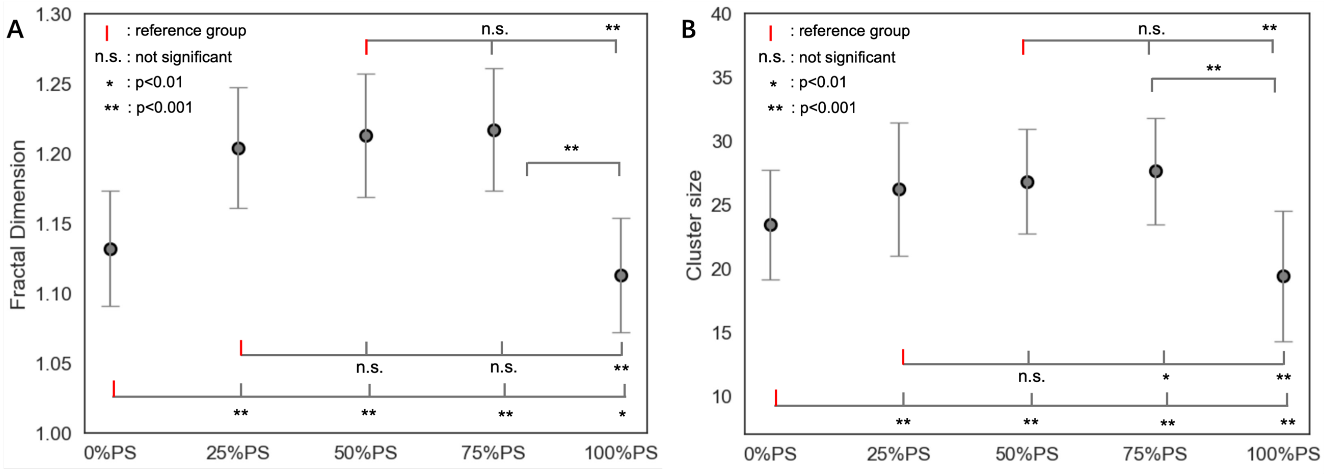

Figure SI. 8: A. Plot of FD of selected MP aggregates with high feature importance (data represents mean ± standard error) vs MP composition type (% PS in PS-PMMA binary mixtures). B. Plot of cluster size of selected MP aggregates (data represents mean ± standard error) vs MP composition type.

SI. 9. Variability in MP particle loading in each TEM grid square for samples before and after different durations of UV exposure

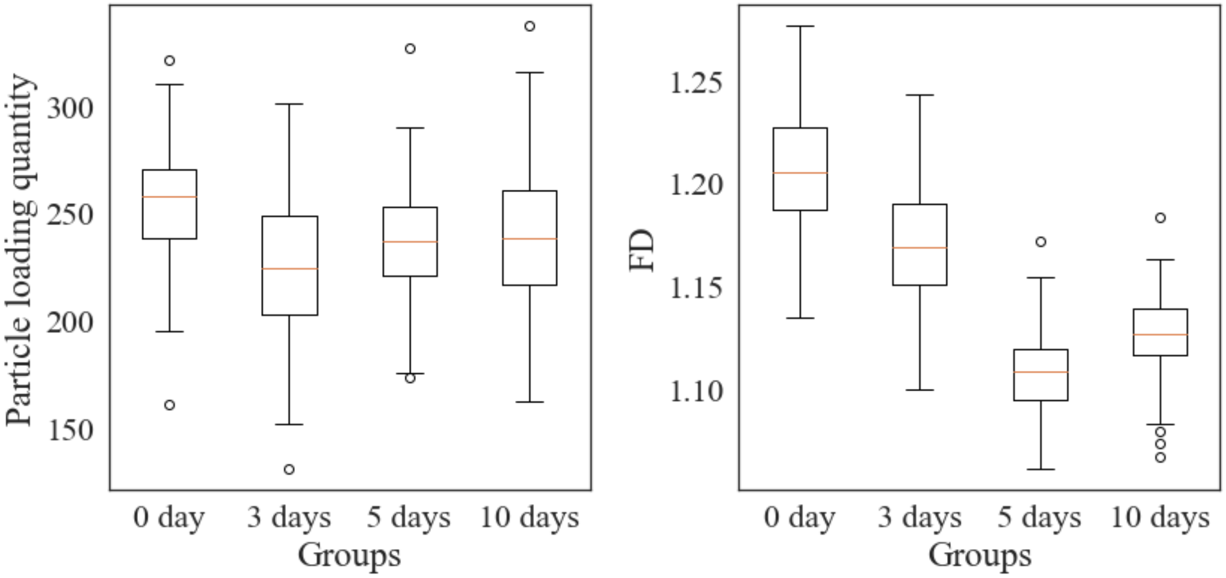

Figure SI. 9: Plot on the left: Analysis of variation in MP loading across single TEM grid squares for the MP sample type of 400 mg/L concentration, with equimolar composition of PS and PMMA, exposed to various durations of UV illumination. Plot on the right shows the observed trend in FD values (Table 2) from 0-10 days of UV illumination for the sample type. This analysis provides further support that the change in FD values reflect changes in MP aggregation behavior and is not introduced from experimental variability in sample loading.

We also investigated the effect of UV exposure on equimolar mixtures of PS and PMMA (Figure 3C). Inspection of Figure 3C and 3F (Table 2) reveals a decrease in both the FD and cluster sizes of MPs on the LC interface with increasing UV exposure times from 0 to 5 days. In the optical micrograph corresponding to 5 days of UV illumination in Figure 3C, the regions corresponding to high feature importance (red regions in the false color micrographs for 5 days UV exposure, in Figure 3C) contain both large aggregates and singly dispersed MPs, with an average FD of 1.11 ± 0.02 (down from 1.21 ± 0.04 prior to UV exposure (Table 2)). However, in the samples illuminated with UV light for 10 days, MP clusters were identified as important features whereas singly dispersed MPs were assigned a lower feature importance (yellow or blue regions). This observation is supported by an increase in FD (1.13 ± 0.02) and cluster size relative to the samples exposed to UV light for 5 days (Table 2). We also analysed the variability in MP loading across our experiments, or the average number density of MPs on the LC interface across each TEM grid square for all PS and PMMA mixture samples before and after UV illumination. We found no significant change in number density of MPs with increase in UV illumination (Figure SI. 9), which suggests that the trends in FD and cluster size reflect UV-induced changes in the LC-mediated interactions between MPs of PMMA.

For equimolar mixtures of PS and PMMA, the overall trend in FD and cluster size with UV exposure (Figure 3F) reflected a trend that is reminiscent of single component PMMA samples (Figure 3D), except that the aggregates with high feature importance for mixed MP samples were on an average, larger than aggregates featured in single-component PMMA samples (Table 2, Figure 3F). Figure 3G shows the classification performance of *DeepPolyNet* on a dataset of 2400 images of aggregates of single component and equimolar mixtures of PS and PMMA MPs, for a single concentration (400 mg/L) (also see Figure SI. 15 and Table S2: single-parameter prediction performance summary for analysis on complete dataset of 8400 images) as a function of UV exposure.

SI. 10. Scheme for analysis of internal organization of PS and PMMA MPs within mixed clusters using fluorescence labelled MPs

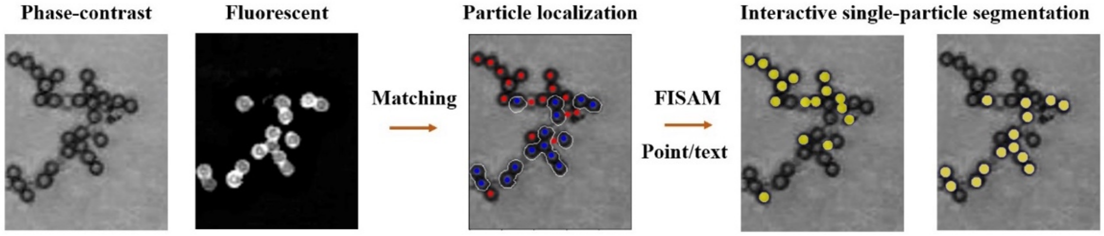

Figure SI. 10: Scheme of analyzing MP binary mixtures using fluorescence labelled particles.

To analyze the internal organization of PS-PMMA MPs within mixture clusters, PS particles were labelled with fluorescent dyes. By overlaying phase-contrast and fluorescence microscopy images, the positions of both PS and PMMA particles were determined. We developed a particle detection and identification algorithm, as shown in Supplementary Figure SI. 10, to identify the position and type of each particle. The algorithm first detects individual particle positions in the phase-contrast images. This process is achieved using segment anything model (SAM) (*80*) with the point and text prompting. Next, it identifies PS particles by thresholding the fluorescent images to isolate non-black regions corresponding to PS particles. By applying this fluorescent mask to the particle detection results, the algorithm accurately determines the position and type of each particle, enabling detailed analysis of binary aggregation. Note that the images in each panel of Figure SI. 10 show different modes of imaging and analysis of the same MP aggregate.

SI. 11. NOM concentration and differential roles of soluble and colloidal NOM components on MP organization

NOM comprises colloidal and soluble components. The concentration of the soluble component of NOM in natural aquatic environments has been measured to range from 0.5-100 mg/L of soluble organic carbon (*81*). We extracted the soluble component of NOM from our peat NOM using a reference method described in a recent publication (*68*). This method of extraction led to a soluble NOM concentration of 30-60 mg/L, which is within the range of NOM concentrations encountered in natural aquatic environments.

The concentration of the colloidal component of NOM in natural aquatic samples ranges from ~2-63 mg/L depending on the sampling location, with lowest levels of colloids measured in forest runoffs and highest in agricultural field or wetlands (*82*). The colloidal component in our peat NOM samples was 79 ± 32 mg/L, as measured from dry sample weight after filtering the NOM solution through a 0.45 µm pore-size filter, which is a concentration comparable to environmental samples with high levels of colloidal NOM.

To provide additional insight into the roles of colloidal and soluble NOM on MP aggregation, we removed the larger colloidal NOM particles from MP samples by filtration (the loading of colloidal NOM was below 1 mg/L) and explored the aggregation behavior of the MPs in the absence of the colloidal NOM. This allowed us to pinpoint the influence of the soluble NOM on the MP aggregation behavior. The results are shown in Figure SI. 11A-C. Inspection of the MP aggregation patterns in the brightfield optical micrographs confirms that the PMMA MPs are more dispersed when incubated in soluble NOM (Figure SI. 11A) relative to PMMA MPs in the absence of NOM (Figure SI. 11B). We also measured PMMA MPs to form larger aggregates (Figure SI. 11C) when both soluble and colloidal NOM components were present (as compared to NOM-free samples; Figure SI. 11B). In particular, as evident in Figure SI. 11C, we observed that MPs accumulated around large colloidal NOM particles.

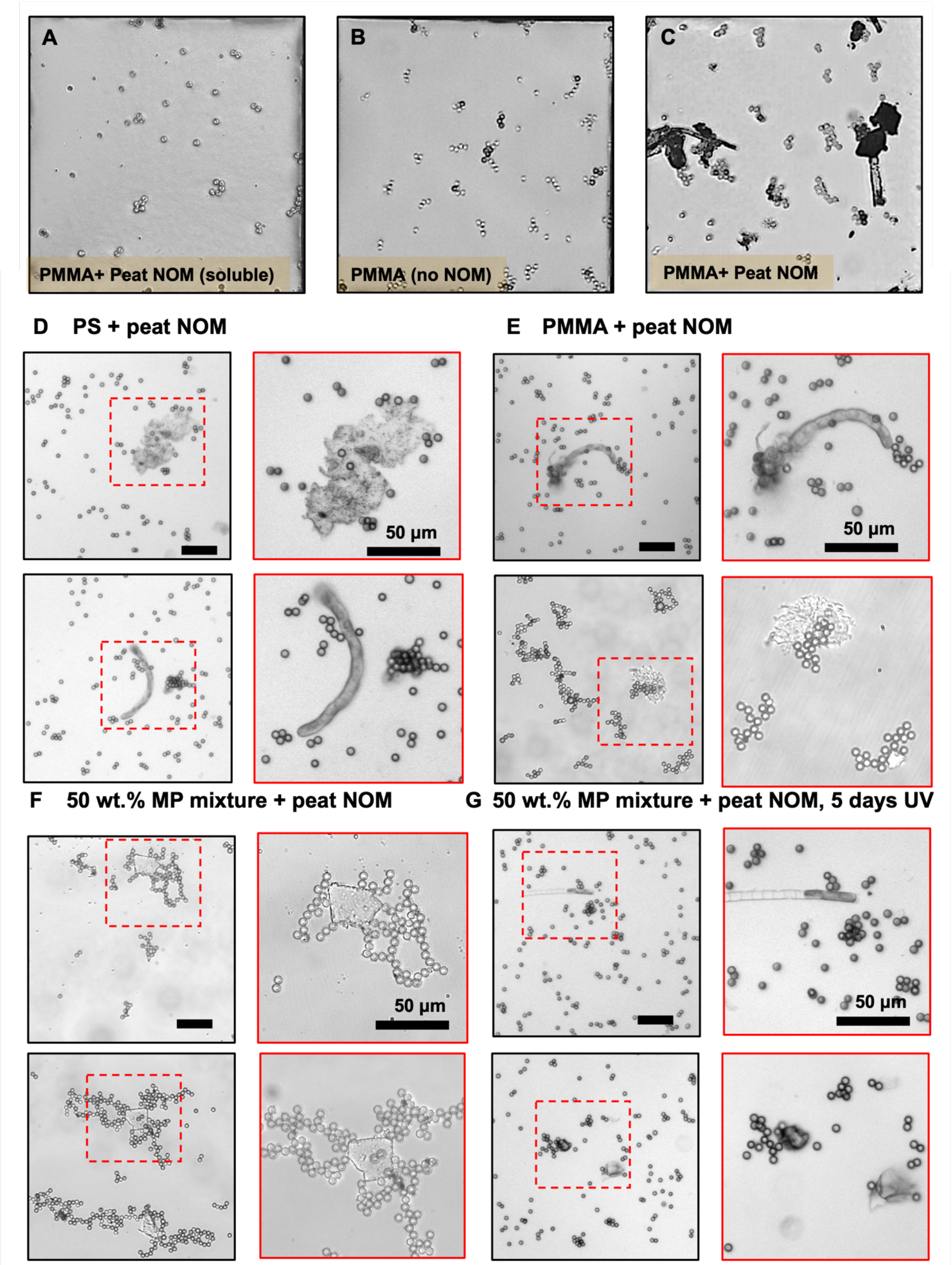

Figure SI. 11: Brightfield optical micrograph of 20 mg/L PMMA MPs incubated at the LC-aqueous interface in (A) the presence of the soluble part of peat NOM, (B) absence of NOM, (C) in the presence of both soluble and colloidal components of peat NOM. Phase contrast optical micrographs showing the influence of NOM (in the presence of both soluble and colloidal components) on organization of single component (D, E) and mixtures of PS and PMMA MPs (F, G) at LC-aqueous interfaces before (F) and after 5 days of aging a 1:1 PS-PMMA MP mixture under 254 nm UV light (G), all in presence of 0.3 M NaCl. The insets show magnified microscopic images of MP clusters adsorbed on to large colloidal NOM particles. The scale bar is 50 µm.

SI.12. Characterization of anchoring of LC on the MP surfaces in the presence of NOM from various sources: in bulk LC

We characterized the anchoring of LC (nematic 5CB) on the surfaces of PMMA MPs (with and without exposure to UV illumination) that had been exposed to the soluble component of NOM from three sources: 1. Peat NOM, 2. Suwannee R. NOM and 3. Mississippi R. NOM (these NOM sources are described in detail in the text accompanying Figure SI. 16). The concentration of soluble NOM in these samples was adjusted to 60 mg/L.

To characterize the anchoring of LC on the MP surfaces in the presence of NOM, we performed two types of experiments: First, we incubated the PMMA MPs in solutions of soluble NOM, dried the MPs and resuspended the MPs in LC. We characterized the orientation of the LC at the MP surfaces using polarized light microscopy, using MPs that had not been exposed to NOM solution as a reference sample (Figure SI. 12A, B). In these experiments, we observed tangential anchoring of LC on the surface of the PMMA MPs (Figure SI. 12C, E, G), which is similar to the control (no NOM). We also repeated these experiments with PMMA MPs that were exposed to 5 days of UV illumination, and the results (Figure SI. 12D, F, H) were also not measurably different from the control (an anchoring transition to a perpendicular orientation was observed to be triggered by UV exposure). Overall, this experiment, which was performed with three sources of NOM, indicates that adsorbed NOM does not cause a change in anchoring of the LC on the MP surface.

Second, because it is possible that removal of the MPs from the NOM solution (as performed in the first experiment described above) may result in the removal of reversibly bound NOM from the MP surfaces, we performed an additional experiment. Specifically, we imaged single MPs of PMMA at the LC-aqueous interface, in the presence of the three NOM sources added to the aqueous phase. In these experiments, to define the azimuthal orientation of the LC phase, we supported the LC on a rubbed polyimide substrate (schematic illustration of director profile in SI. 13I-L). Again, in these experiments, when soluble NOM was present in the aqueous phase, we observed tangential anchoring of LC on the surface of the PMMA MPs (Figure SI. 13C, E, G) which is the same LC orientation observed before addition of the soluble NOM (Figure SI. 13A). We also repeated these experiments with PMMA MPs that were exposed to 5 days of UV illumination (Figure SI. 13D, F, H). In these experiments, the anchoring of the LC on the MPs in the presence of the NOM was also the same as that observed on MPs that had not been exposed to the NOM (Figure SI. 13B, perpendicular anchoring of the LC after 5 days of UV exposure).

Overall, these experiments show that the LC orientation does not change when PMMA MPs were exposed to three different sources of soluble NOM. For PMMA samples subjected to 5 days of UV light treatment prior to exposure to the soluble NOM, we observed the LC on the PMMA to exhibit a perpendicular orientation, similar to our observation of LC anchoring (Figure 8) in the absence of NOM.

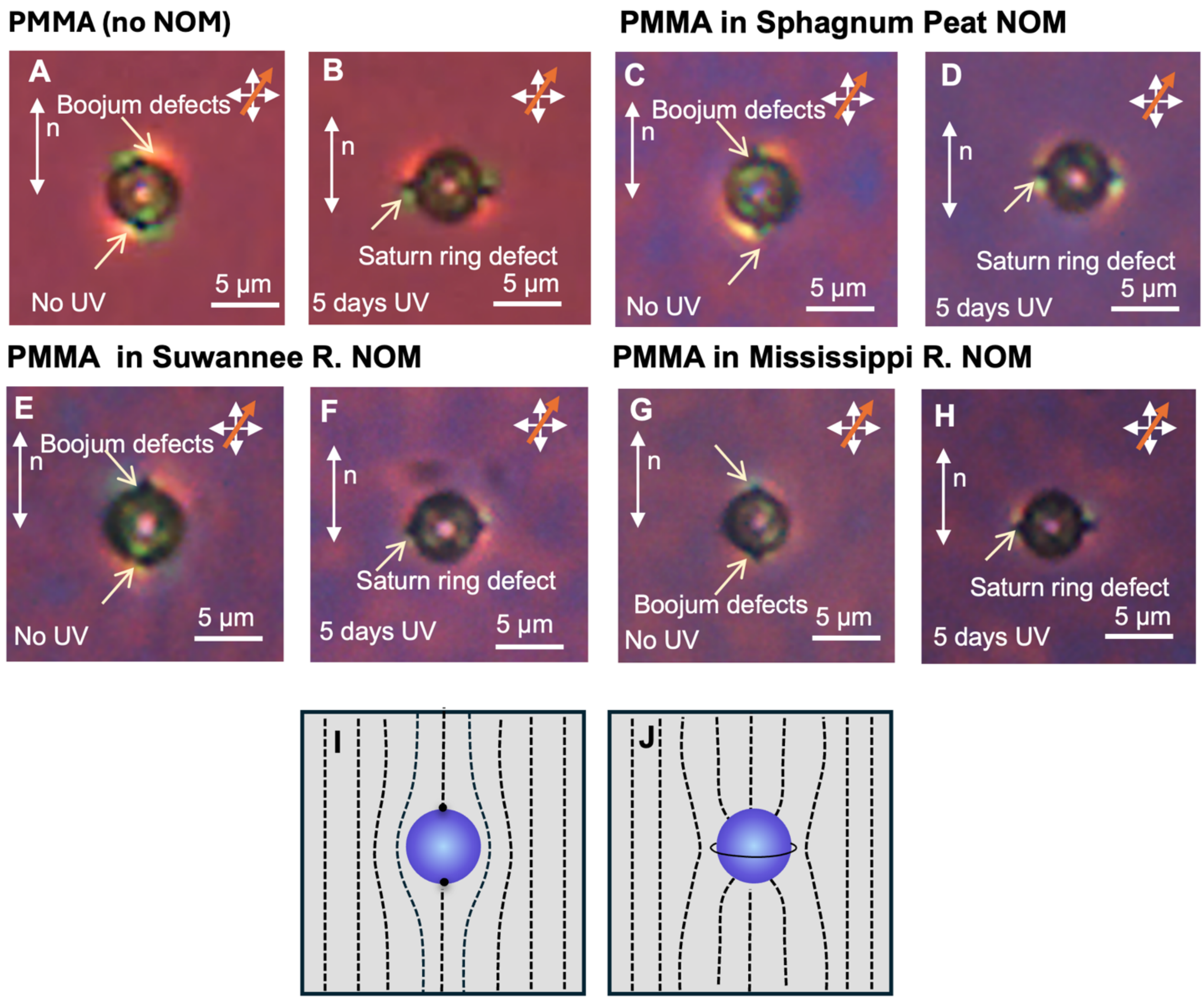

Figure SI. 12: Single PMMA MPs redispersed in LC after being incubated in aqueous solutions of soluble NOM from three different sources (peat, Suwannee R. and Mississippi R.). (A, B) Control experiment without NOM: PMMA MP before (A) and after 5 days of exposure to 254 nm UV light (B). (C, D) A single PMMA MP incubated in soluble peat NOM before (C) and after 5 days of exposure to 254 nm UV light (D). (E, F) A single PMMA MP incubated in Suwannee R. NOM before (E) and after 5 days of exposure to 254 nm UV light (F). (G, H) A single PMMA MP incubated in Mississippi R. NOM before (G) and after 5 days of exposure to 254 nm UV light (H). Schematic illustration of a single PMMA MP before (I) and after 5 days of exposure to UV light (J). The black dashed lines indicate the LC director profile. The optical micrographs show optical images obtained using polarized light microscopy (crossed polarizers) using a retardation plate (λ-plate (530 nm)) inserted at 45° with respect to the polariser in an optical cell with a thickness of 27-32 µm and planar anchoring (see fabrication of bulk optical cells in Supplementary Methods and Materials). Note that Figure SI. 12 shows results obtained over a much broader range of experimental conditions than those in Figure 8D and F (main text). The images shown in Figures 8D and F (main text) are duplicated in Figure SI. 12A and B to serve as references for Figure SI. 12C-H. Additionally, optical micrograph in Figure SI. 12B is reused in Figure SI. 18C to facilitate a comparison of the presented data.

SI. 13. Characterization of anchoring of LC on the MP surfaces in the presence of NOM from various sources: at LC-aqueous interface

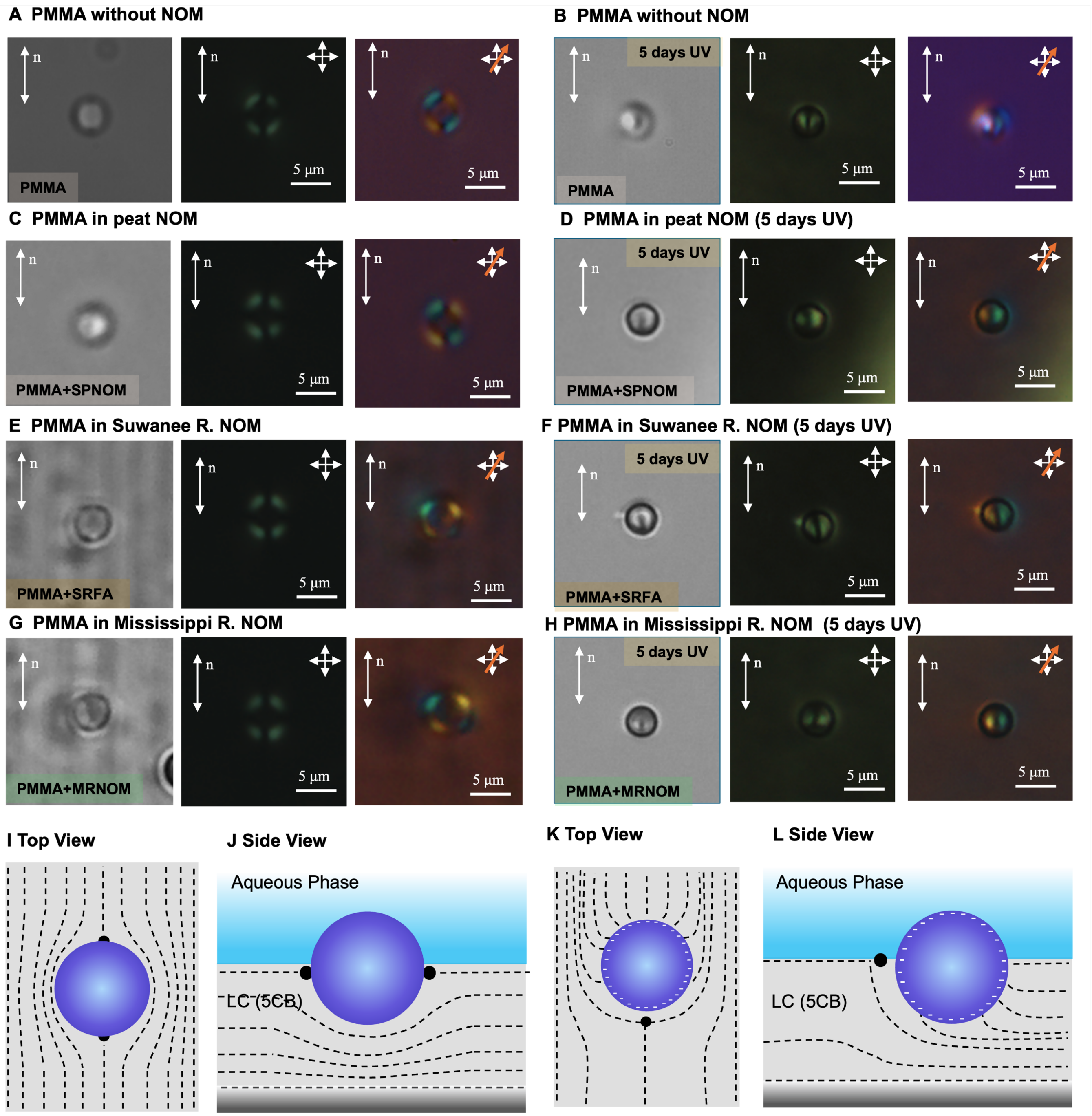

Figure SI. 13: Optical micrographs obtained using brightfield (left or first column) and polarized light microscopy (crossed polarizers) without (middle or second column) and with a retardation plate (λ-plate (530 nm)) inserted at 45° with respect to the polarizer (right or third column). The optical micrographs show single PMMA MPs from samples incubated: (A) in dark for 5 days in 0.3M NaCl (without NOM),

(B) under UV light exposure for 5 days; (C) in dark for 5 days in the presence of peat NOM, (D) in presence of peat NOM and UV light exposure for 5 days; (E) in dark for 5 days in the presence of Suwannee R. NOM, (F) in presence of Suwannee R. NOM and UV light exposure for 5 days. (G) in dark for 5 days in the presence of Mississippi R. NOM, (H) in presence of Mississippi R. NOM and UV light exposure for 5 days. The quadrupolar symmetry of the optical textures, evident in the cross-polar optical micrographs in A, C, E, G (images in second and third columns), is consistent with tangential anchoring of LC around the PMMA MP. The dipolar symmetry of the optical texture evident in the cross-polar images in B, D, F, H (images in second and third columns) is consistent with perpendicular anchoring of LC around the UV exposed PMMA MP. Schematic illustration of top view (left) and side view (right) of PMMA MP at LC-aqueous interface with the LC film supported on a substrate that causes planar anchoring. The anchoring of LC on the surface of the MP is shown as tangential (I, J) or radial, (K, L).

SI. 14. Colloidal NOM and MP heteroaggregation in bulk aqueous phase

Because both the MPs and colloidal NOM possess negative surface charge densities(*83*, *84*), charge-mediated interactions are unlikely to be responsible for driving heteroaggregation. Additionally, MPs and NOM aggregate in bulk aqueous solution, indicating that LC-mediated interactions are not needed to drive heteroaggregation (Figure SI. 14A-D). Given the heterogeneous nature of NOM, heteroaggregation with MPs is likely to reflect a range of interactions, including hydrophobic, van der Waals interactions.

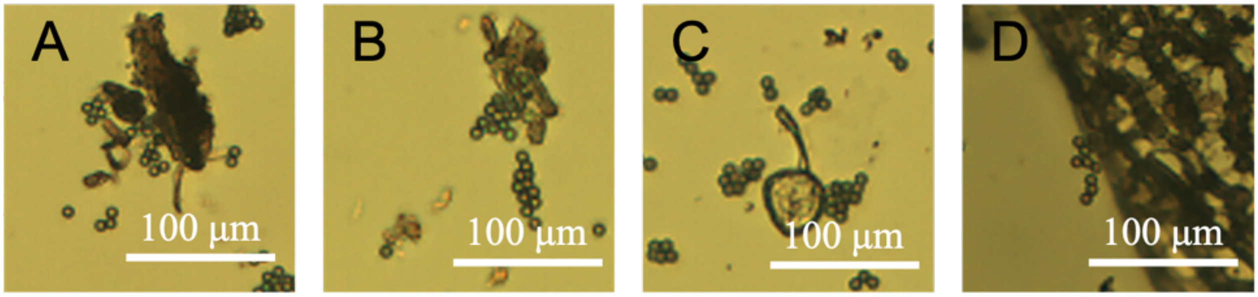

Figure SI. 14: Optical micrographs of heteroaggregates of colloidal NOM and an equimolar PS and PMMA mixture of MPs dispersed in bulk aqueous phase.

SI. 15. Ability of *DeepPolyNet* to correctly recognize MP samples within 42 sample types

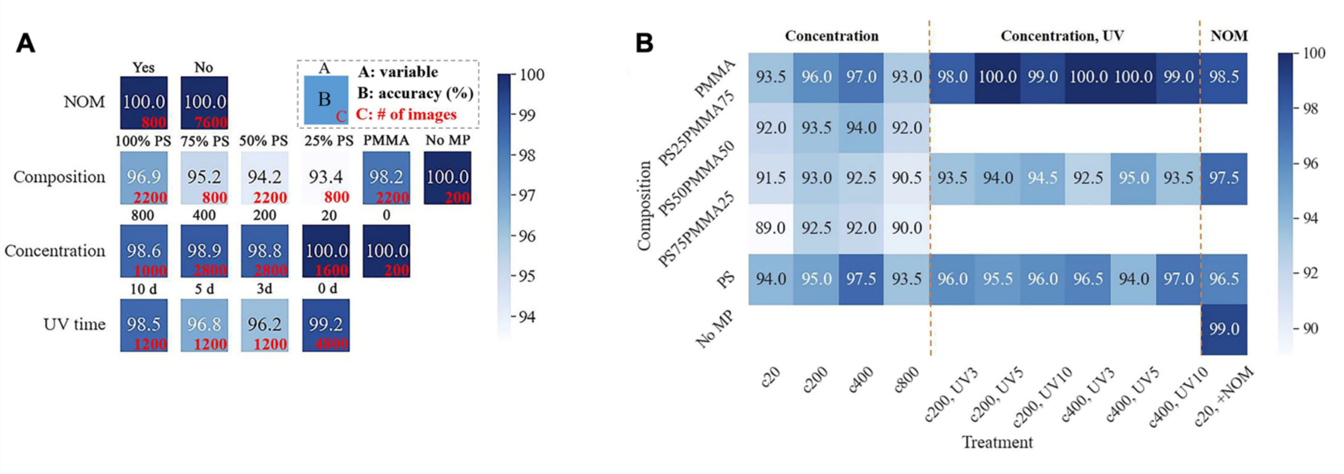

Figure SI. 15: (A) Accuracy of single-parameter recognition summary of *DeepPolyNet*. This table shows the sample types (A: variable or sample type), the recognition accuracy of that particular sample type by the *DeepPolyNet* algorithm (B), and the corresponding total number of images used per category (C: divided into training (70%), validation (20%) and testing (10%)). (B) The confusion matrix illustrates the multi-parameter prediction accuracy for classifying optical micrographs into one of the 42 sample types, where accurate predictions require correct identification of sample types within all four categories.

We summarize the results for accurate recognition of single parameters (e.g. composition type) for all images in the dataset (Figure SI. 15A). While the overall accuracy is high as compared to Figure SI. 15B, the challenge still lies in predicting sample composition, especially for mixed MP samples. The prediction accuracies for 75% PS (25% PMMA), 50% PS and 25% PS are 95.2%, 94.2% and 93.4% respectively, while that of 100% PS and 100% PMMA are 96.9%, 98.2% respectively, where incorrect prediction corresponds to assignment of an input image to one of the composition sample types different from its ground truth. Figure SI. 15B is discussed in the main text.

SI. 16. Evaluating *DeepPolyNet* for classification of samples with a wider range of colloidal NOM concentrations and NOM types

We evaluated the ability of *DeepPolyNet* to classify MP samples that contained NOM from three different sources. In addition to peat NOM described in the main text, we obtained two different NOM sources from the International Humic Substances Society (IHSS): 1. Suwannee River Fulvic Acid (Suwannee R. NOM), and 2. Upper Mississippi River Natural Organic Matter (Mississippi R. NOM). We found the concentration of colloidal NOM (> 0.45 µm) in the Suwannee R. and Mississippi R. samples to be <1mg/L which is at lower end of colloidal NOM concentrations found in environmental samples.

In order to classify the MP samples containing varied NOM sources, we augmented the dataset used to train *DeepPolyNet* to include samples that covered the environmentally relevant range of colloidal NOM concentrations. Specifically, the training dataset contained 20 mg/L MP samples (PS or PMMA) with peat NOM (not filtered; colloidal NOM concentration of 79 ± 32 mg/L) and filtered peat NOM (colloidal NOM concentration <1 mg/L). We also included the same NOM samples without MPs in the training dataset. Briefly, for samples containing both MPs and NOM, the algorithm was able to classify the sample MP

composition (i.e., the presence of PS only, PMMA only, or a mixture of PS and PMMA) with high accuracy (>95%) (Figure SI. 16A). The inclusion of the broader range of colloidal NOM concentrations in the dataset (specifically samples with low colloidal NOM concentrations) led to only a small (<1-4%) decrease in classification accuracy (compare to Figure 5F). This decrease is ascribed to the absence of large colloidal particulates in the filtered peat NOM, which are easily distinguished as NOM.

Next, we analysed the sample recognition performance of *DeepPolyNet* (after retraining as described above) through "blind tests" of MP samples containing the three NOM sources. The prediction accuracies are reported in Figure SI. 16B and represent the average values from "blind testing" of 50 sample images in each category, where each correct prediction corresponds to accurate classification of all 4 sample parameters (i.e., concentration, composition, presence of NOM, UV aging duration). The recognition accuracies for the samples containing the new NOM sources are lower than those obtained previously using peat NOM only (84-90% for MPs incubated in the two NOM sources versus 90-92% for MPs incubated in peat NOM only), a result that suggests that different sources of NOM do have differential effects on MP aggregation. From these experiments, we conclude also that additional training of the algorithm on samples containing diverse sources of NOM will likely improve the sample recognition capability of our algorithm.

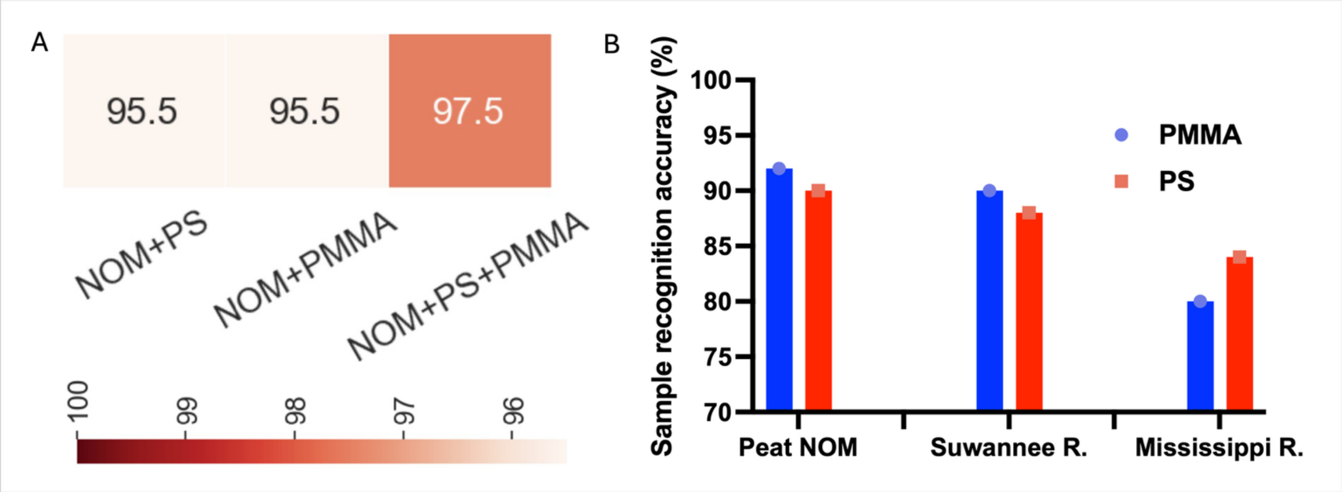

Figure SI. 16: (A) Results of *DeepPolyNet* classification after retraining the CNN with images of PS and PMMA MPs incubated in the soluble component of peat NOM. The numbers within the boxes indicate sample recognition accuracies using samples that contained peat NOM (with and without colloidal NOM). (B) Bar plot of sample recognition accuracy for 50 "blind" samples of PMMA (blue) and PS (red) MPs incubated in Peat NOM, Suwannee R. NOM and Mississippi R. NOM (no UV exposure) after retraining *DeepPolyNet* as described in A. An accurate prediction corresponds to correct classification of all 4 sample parameters (i.e., concentration, composition, presence of NOM, UV aging duration).

SI. 17. Anchoring transition of 5CB on PMMA MP surface

Past studies have shown that 5CB anchors on PS and PMMA polymer films with tangential orientation, a conclusion that we confirmed for the MPs used in our experiments. For this experiment, we dispersed dry PS and PMMA MPs (5 μm diameter) in bulk 5CB and observed them in an optical cell (27-32 μm thickness) with uniform planar alignment. We observed surface or point defects, so-called "boojum" defects (Figure 8C, D and G, H) at the north and south poles of individual particles for both PMMA and

PS, consistent with a quadrupolar configuration and planar anchoring of 5CB on both the MP surfaces before exposure to UV light.

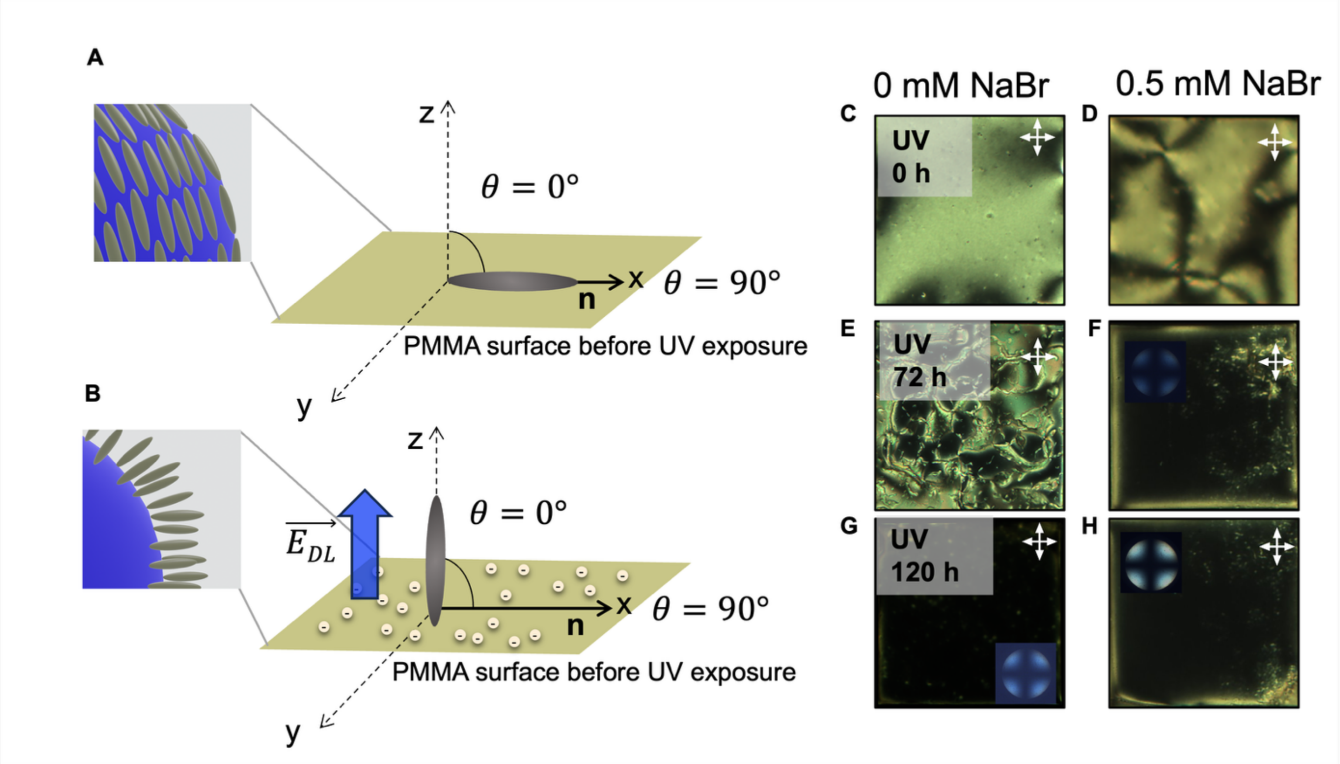

Figure SI. 17: (A, B) Schematic illustration showing the coupling of electric double layer to LC orientation on PMMA surface. (A) shows the surface of PMMA prior to UV exposure, where the orientation of LC mesogens on PMMA surface is tangential, (B) shows the surface of PMMA after UV exposure. The surface has a high negative charge density with the orientation of LC mesogens on PMMA surface being perpendicular. (C-H) Cross-polarized optical micrographs of 5CB (C, E, G) and 0.5 mM NaBr doped 5CB (D, F, H) on PMMA surface exposed to different durations of 254 nm UV light. Each grid square represented in c-h is 284 x 284 μm^2.

After 5 days of UV illumination, about 70% of the irradiated PMMA samples exhibited a line or a Saturn ring defect (quadrupolar symmetry; Figure 8E, F), indicating perpendicular anchoring of 5CB on PMMA surface. In contrast, 5CB anchoring on PS surface remained tangential (Figure 8I, J). We further confirmed this observation by exposing thin films of PMMA to similar durations of UV light and observing LC anchoring using polarized light microscopy (crossed polars). Before UV exposure, 5CB exhibited tangential anchoring on PMMA, transitioning to homeotropic anchoring after 120 hours of UV exposure. Close inspection of the optical micrograph in Figure SI. 17E reveals that the transition started with the formation of homeotropic domains, indicating chemical changes on the PMMA surface leading to patchy domains that promote homeotropic anchoring of 5CB.

Past studies have shown that the LC orientation for LCs with positive dielectric anisotropy, such as 5CB ($\Delta\varepsilon=\varepsilon_{\|}-\varepsilon_{\perp}\sim 10$, for 5CB) are coupled to electric double layers formed on charged surfaces, and promote homeotropic anchoring. Briefly, because the LC possesses anisotropic dielectric properties, the electric field within the electrical double layer induces a torque on the mesogens thereby causing the LC mesogens to orient with its largest dielectric constant oriented parallel to the lines of the electric field. The properties

of the electric double layer, the Debye length, also impacts the orientation of the LC. Shah et. al. (*71*), showed that in carboxylic acid terminated self-assembled monolayers of alkane thiol ($HOOC(CH_2)_{10}SH$), pretreated at pH 10.6, homeotropic anchoring was promoted at high ionic strength due to an increase in the electrostatic contribution to the anchoring energy. We hypothesized that the anchoring transition from tangential to radial on PMMA after UV exposure is due to the charging of PMMA surface. To confirm this, we doped 5CB with various concentrations of electrolyte (NaBr). Doped samples showed an early onset of anchoring transition (homeotropic anchoring after 72 h UV exposure for 0.5 mM NaBr (Figure SI. 17F), compared to 120 h for undoped 5CB (Figure SI. 17G).), consistent with electrostatic contributions to anchoring energy, which is predicted to increase with decreasing Debye length for an approximately constant potential surface behavior.

SI. 18. Characterization of LC anchoring around UV-exposed PMMA MPs for a LC with a negative dielectric anisotropy

As described in the main text, in contrast to 5CB, we found that UV-treated PMMA particles did not cause the nematic MBBA to adopt a radial alignment at the MP surface (Figure SI. 18).

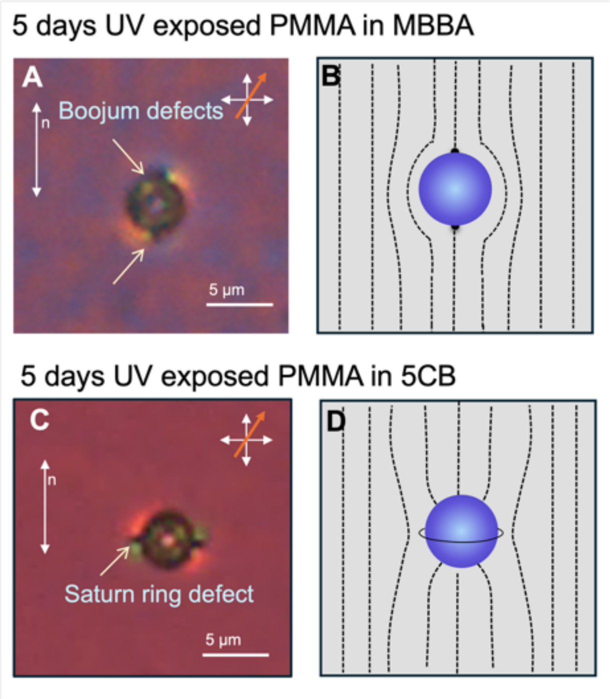

Figure SI. 18: A, B. Optical micrograph and schematic illustration of a single PMMA MP exposed to UV for 5 days. The MP was dispersed within a 27-32 µm-thick film of MBBA with planar anchoring. C, D. Optical micrograph and schematic illustration of a single PMMA MP exposed to UV for 5 days. The MP was dispersed within a 27-32 µm-thick film of 5CB with planar anchoring (also shown in Figure 8). The black dashed lines indicate the LC director profile. The optical micrographs indicate optical textures from cross-polarized microscopy using a retardation plate (λ-plate (530 nm)) at 45° with respect to the polarizer. Figure SI. 18C is duplicated in Figure SI. 12B and Figure 8F (main text) to facilitate comparison of the data.

SI. 19. Water contact angle measurements on PMMA films for different durations of UV exposure

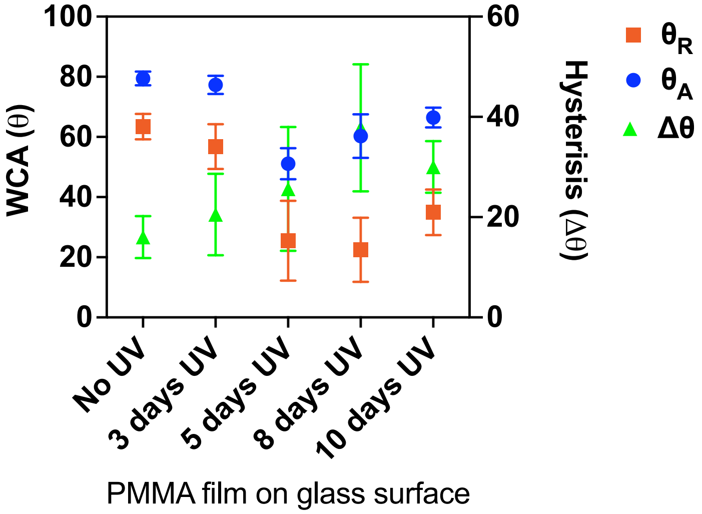

Figure SI. 19: Advancing and receding contact angles of water measured on PMMA surfaces exposed to UV light for the indicated durations. The plot shows mean ± standard deviation for advancing (θ_A) and receding contact angles (θ_R), as well as the contact angle hysteresis, ($\Delta\theta=\theta_A-\theta_R$).

We measured the advancing and receding contact angles of water on UV-treated PMMA and observed that the generation of carboxylate anions on the surface of the UV-treated PMMA leads to a change in water contact angle. We found that the contact angle of water on PMMA films decreased with up to 5 days of UV exposure, reaching a minima, followed by an increase in contact angle for 8 and 10 days of UV exposure. This non-monotonic behavior is consistent with changes in the surface density of carboxylate ions on the surface of the PMMA (as determined by PMIRRAS measurement (Figure 8A) and zeta potential measurements (see Figure 8B). In particular, the less intuitive decrease in density of carboxylate ions with long UV exposure times has been reported to occur via decarboxylation reactions(*54*, *85*).

SI. 20. Evidence of MP chaining after prolonged incubation of UV-exposed PMMA MP samples at LC-aqueous interface

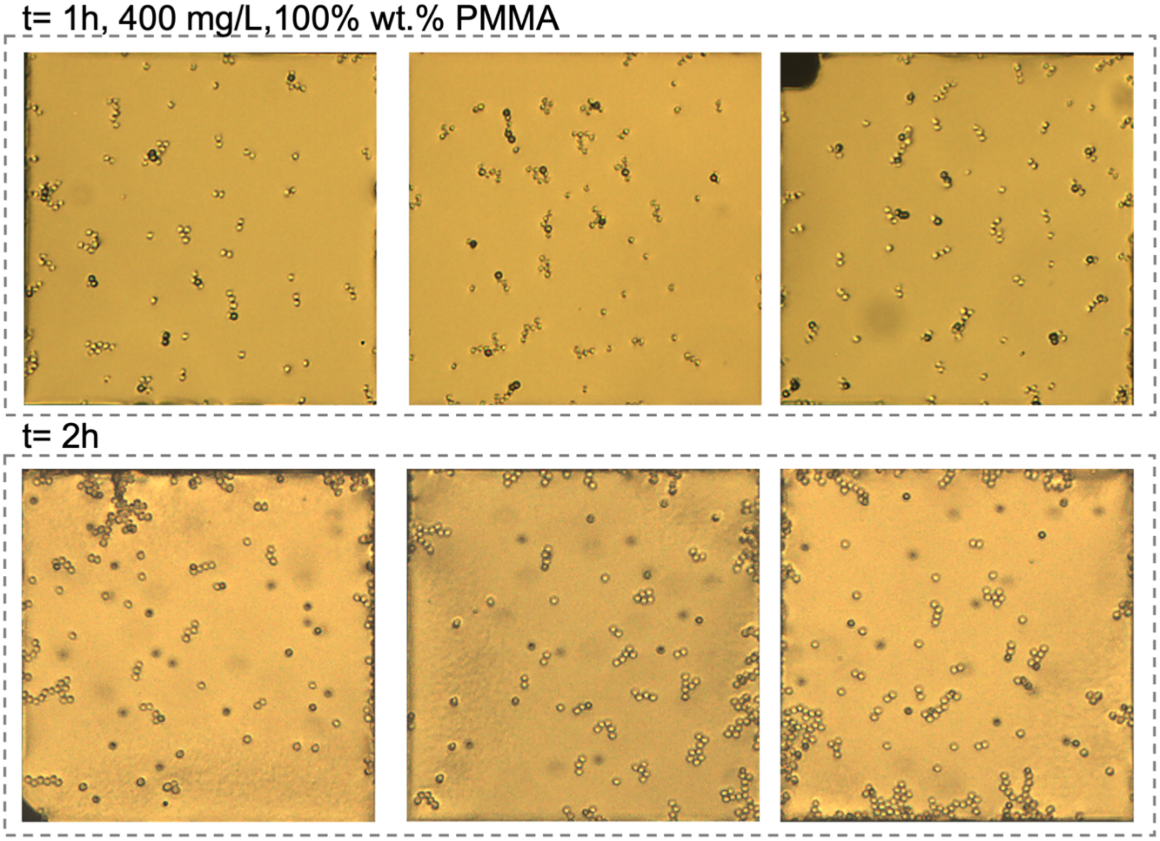

Figure SI. 20: Brightfield optical micrographs of single component PMMA MPs showing evidence of MP chaining when the MP incubation times at LC-interface was extended beyond 15 minutes to 1h (top row) and 2h (bottom row). Each grid square is 284 x 284 μm^2.

Table S3. Sample recognition performance of *DeepPolyNet* using multiple grid squares as input

We re-defined our input samples to be collection of images of 4-6 grids squares from a single TEM grid incubated under the same unknown aqueous MP sample (instead of using a single grid square). Subsequently, the sample type prediction was chosen as the most frequently predicted set of sample attributes across the collection of individual grid squares within each input sample.

Sample type	Accuracy of single grid squares as input images	Accuracy of 4-6 grids squares as input images	Total number of grid squares tested
c20, PS75PMMA25, No UV, No NOM	75%	9/10	44
c800, PS75PMMA25, No UV, No NOM	83%	10/10	46
c800, PS50PMMA50, No UV, No NOM	86%	10/10	42
c200, PS50PMMA50, No UV, No NOM	93%	10/10	45
c400, PS50PMMA50, UV3, No NOM	90%	10/10	51
c20, PS, No UV, +NOM	92%	10/10	53
c400, PS50PMMA50, UV10, No NOM	92%	10/10	49

Supplementary Methods and Materials

Materials

Chemicals were used as purchased from manufacturers without further purification. 5CB (>99.5%) was purchased from Jiangsu Hecheng Advanced Materials Co. Ltd. (Nanjing, China). NaCl (>99%), Rhodamine B (>95%, powder) was purchased from Sigma Aldrich. PS and PMMA 5 μm particles (10 mg/mL) were purchased from Alpha Nanotech Inc. Suwannee River Fulvic Acid (Suwannee R. NOM) and Upper Mississippi River Natural Organic Matter NOM (Mississippi R. NOM) standards were purchased from International Humic Substances Society (IHSS). Deionization of a distilled water source was performed using a distilled and deionized water (resistivity of more than 18.2 MΩ) Milli-Q system (Millipore, Bedford, MA). 20μm-thick copper transmission electron microscopy (TEM) grids were purchased from Electron Microscopy Sciences. Polyimide solutions were purchased from HD Microsystems (Parlin, NJ) and were used to prepare slides for optical cells by spin-coating them on glass slides as per the manufacturer's instructions.

Spin coating thin films of PMMA

Powdered PMMA was dissolved in toluene at a concentration of 0.2 wt.%. The PMMA solution was then spin coated onto a gold-coated Si-wafer substrate at 1000 to 3000 rpm for 1 minute. The acceleration and deceleration to and from the desired spin speed occurred in 5 seconds. The samples were then cured at room temperature overnight and then in an oven for 2 hours at 90 °C.

Preparation of UV exposed PS and PMMA MPs for characterization in bulk LC
Aqueous dispersions of MPs in 0.3 M NaCl were exposed to UV irradiation for 5 days, followed by rinsing three times with water and ethanol. Subsequently, the microparticles were dried and redispersed in bulk 5CB for analysis shown in Figure 8. The UV lamps were switched off every 12 h, for a duration of 2h.

Fabrication of Millifluidic Sensing Device
Figure SI. 1 shows a schematic illustration of the millifluidic channel used in our experiments. The millifluidic channel comprises three layers. The top two layers are made of acrylic sheet (1.5 mm thickness from ZLazr.com, USA), laser-cut into uniform dimension (75x26 mm) of a standard glass slide, and the bottom layer was an OTS treated glass microscope slide as described above. The two top layers were permanently bonded by applying chloroform to the contacting surfaces of the acrylic sheets and rested overnight. This was followed by a thorough cleaning step with sequential immersion of the acrylic sheet layers in water, ethanol and hexane. Residual solvents were dried off under a steam of gaseous N_2.

Preparation of Thin LC Films
The LC used in our study was 4-pentyl-4′cyanobiphenyl (5CB) (HCCH, Jiangsu Hecheng Display Technology Co., Ltd.), a thermotropic LC that forms a nematic LC between 24 and 35 °C. 20 µm-thick LC films were prepared by pipetting 0.5 µL of 5CB into the pores of 75 mesh (thickness 20 µm; lateral pore size 284 µm) transmission electron microscopy (TEM) grids that were supported on OTS-treated glass slides. Excess LC was then removed by a micropipette tip using capillary action to produce a flat LC film with a thickness of 20 µm.

Fluorescence Labelling of polystyrene (PS) MPs
Fluorescent labeling of 5 µm polystyrene (PS) particles was performed using Rhodamine B dye. The dye was initially prepared at a concentration of $5x10^{-4}$ mol/L in tetrahydrofuran (THF). Briefly, we introduced 100 µL of the dye solution to 600 µL of an aqueous PS suspension (0.5 w%). The mixture was incubated for 1 hour at room temperature, followed by the addition of 800 µL of water. During the incubation, the suspension was constantly agitated in a shaker plate at 30 rpm and subsequently centrifuged at 5000 g for 10 min. After centrifugation, the particles were washed twice with distilled water, air-dried and re-suspended in 0.3 M aqueous NaCl to desired concentrations.

Optical and Fluorescence Microscopy
Brightfield and cross-polarized imaging was carried out using an Olympus BX41 microscope equipped with 10×, 20× objectives, two rotating polarizers, and a Moticam 10.0 MP camera. Fluorescence and phase contrast imaging was done on an inverted epifluorescence microscope (IX81, Olympus Life Science) using a 20× water immersion objective.

Preparation of MP solution
The PS and PMMA MPs were cleaned by centrifuging for 5 minutes, a 1 mg/mL particle solution in Milli-Q water at 3000 rpm, for three times, followed by drying at room temperature. Measured amounts of MPs were then dispersed in 0.3M aqueous NaCl followed by sonication and vortexing to obtain dispersions with singly dispersed MPs in aqueous solution. The prepared MP dispersions were introduced into the millifluidic channel through the inlet using a glass pipette.

Fabrication of optical cells for bulk LC observations

The setup for MP characterization in bulk 5CB involved homogeneously planar aligned sandwich cells with a constant gap of 27-32 μm. The top and bottom substrates were rubbed polyimide coated and were assembled antiparallelly. 5CB was heated to isotropic phase and a few particles were mixed with bulk 5CB. This was then used to fill the sandwich cell, incubated at 60°C, still in isotropic phase to avoid flow induced LC alignments. The individual particles were observed under the optical microscope as 5CB cooled down to nematic phase.

Zeta Potential Measurement

Microparticle dispersions in water was formed using DI water comprising in the presence of NaCl. After allowing 30 min for the sample to equilibrate, ζ on the aqueous side of the microparticles was measured using the Malvern Zetasizer Nano instrument.

Fourier-Transformed Polarization-Modulation Infrared Reflectance Absorbance Spectroscopy (PM-IRRAS)

Substrates used in the IR measurements were prepared by sequential deposition of 20 Å of Ti and 200 Å of Au onto Si wafers using an electron beam evaporator. Films of PMMA were deposited on top of the Au films by spin coating from a 0.2 wt.% PMMA solution in toluene. IR spectra of the PMMA polymer film before UV irradiation and after different durations of UV irradiation were obtained using a Nicolet Magna-IR 860 FT-IR spectrometer with a photoelastic modulator (PEM-90, Hinds Instruments), synchronous sampling demodulator (SSD-100), and a liquid N_2-cooled mercury cadmium telluride (MCT) detector. All spectra (700–4000 cm^{-1}) were recorded at an incident angle of 83° with the modulation centered at 1500 cm^{-1}. For each sample, 1000 scans were taken at a resolution of 4 cm^{-1}. Data were collected as differential reflectance vs wavenumber. All IR results presented were analyzed by OMNIC software.

REFERENCES AND NOTES

1. R. C. Thompson, W. Courtene-Jones, J. Boucher, S. Pahl, K. Raubenheimer, A. A. Koelmans, Twenty years of microplastic pollution research—What have we learned? *Science* **386**, eadl2746 (2024).

2. J. A. I. do Sul, M. F. Costa, The present and future of microplastic pollution in the marine environment. *Environ. Pollut.* **185**, 352–364 (2014).

3. C. Xu, B. Zhang, C. Gu, C. Shen, S. Yin, M. Aamir, F. Li, Are we underestimating the sources of microplastic pollution in terrestrial environment? *J. Hazard. Mater.* **400**, 123228 (2020).

4. J. D. Drummond, U. Schneidewind, A. Li, T. J. Hoellein, S. Krause, A. I. Packman, Microplastic accumulation in riverbed sediment via hyporheic exchange from headwaters to mainstems. *Sci. Adv.* **8**, eabi9305 (2022).

5. N. Weithmann, J. N. Möller, M. G. J. Löder, S. Piehl, C. Laforsch, R. Freitag, Organic fertilizer as a vehicle for the entry of microplastic into the environment. *Sci. Adv.* **4**, eaap8060 (2018).

6. R. S. Bang, M. Bergman, T. Li, F. Mukherjee, A. S. Alshehri, N. L. Abbott, N. C. Crook, O. D. Velev, C. K. Hall, F. You, An integrated chemical engineering approach to understanding microplastics. *AIChE J.* **69**, e18020 (2023).

7. I. Ali, X. Tan, J. Li, C. Peng, I. Naz, Z. Duan, Y. Ruan, Interaction of microplastics and nanoplastics with natural organic matter (NOM) and the impact of NOM on the sorption behavior of anthropogenic contaminants—A critical review. *J. Clean. Prod.* **376**, 134314 (2022).

8. A. M. Elert, R. Becker, E. Duemichen, P. Eisentraut, J. Falkenhagen, H. Sturm, U. Braun, Comparison of different methods for MP detection: What can we learn from them, and why asking the right question before measurements matters? *Environ. Pollut.* **231**, 1256–1264 (2017).

9. M. Claessens, L. Van Cauwenberghe, M. B. Vandegehuchte, C. R. Janssen, New techniques for the detection of microplastics in sediments and field collected organisms. *Mar. Pollut. Bull.* **70**, 227–233 (2013).

10. M. Eriksen, S. Mason, S. Wilson, C. Box, A. Zellers, W. Edwards, H. Farley, S. Amato, Microplastic pollution in the surface waters of the Laurentian Great Lakes. *Mar. Pollut. Bull.* **77**, 177–182 (2013).

11. R. N. Cable, D. Beletsky, R. Beletsky, K. Wigginton, B. W. Locke, M. B. Duhaime, Distribution and modeled transport of plastic pollution in the Great Lakes, the world's largest freshwater resource. *Front. Environ. Sci.* **5**, doi.org/10.3389/fenvs.2017.00045 (2017).

12. S. L. Wright, J. M. Levermore, F. J. Kelly, Raman spectral imaging for the detection of inhalable microplastics in ambient particulate matter samples. *Environ. Sci. Technol.* **53**, 8947–8956 (2019).

13. G. Erni-Cassola, M. I. Gibson, R. C. Thompson, J. A. Christie-Oleza, Lost, but found with Nile red: A novel method for detecting and quantifying small microplastics (1 mm to 20 μm) in environmental samples. *Environ. Sci. Technol.* **51**, 13641–13648 (2017).

14. B. C. Colson, A. P. M. Michel, Flow-through quantification of microplastics using impedance spectroscopy. *ACS Sens.* **6**, 238–244 (2021).

15. S. Han, J. Bang, D. Choi, J. Hwang, T. Kim, Y. Oh, Y. Hwang, J. Choi, J. Hong, Surface pattern analysis of microplastics and their impact on human-derived cells. *ACS Appl. Polym. Mater.* **2**, 4541–4550 (2020).

16. S. E. Nelms, E. Easman, N. Anderson, M. Berg, S. Coates, A. Crosby, S. Eisfeld-Pierantonio, L. Eyles, T. Flux, E. Gilford, C. Giner, J. Hamlet, N. Hembrow, J. Hickie, P. Hopkinson, D. Jarvis, J. Kearsley, J. Millard, F. Nunn, E. Pollitt, A. Sainsbury, S. Sayer, R. Sinclair, A. Slack, P. Smith, R. Thomas, J. Tyler, R. Walker, C. Wallerstein, M. Ward, B. J. Godley, The role of citizen science in addressing plastic pollution: Challenges and opportunities. *Environ. Sci. Policy* **128**, 14–23 (2022).

17. B. Jorgensen, M. Krasny, J. Baztan, Volunteer beach cleanups: Civic environmental stewardship combating global plastic pollution. *Sustain. Sci.* **16**, 153–167 (2021).

18. F. M. Hecht, A. R. Bausch, Kinetically guided colloidal structure formation. *Proc. Natl. Acad. Sci. U.S.A.* **113**, 8577–8582 (2016).

19. F. Camerin, E. Zaccarelli, Soft colloids for complex interfacial assemblies. *Proc. Natl. Acad. Sci. U.S.A.* **119**, e2122051119 (2022).

20. M. Liu, X. Zheng, V. Grebe, D. J. Pine, M. Weck, Tunable assembly of hybrid colloids induced by regioselective depletion. *Nat. Mater.* **19**, 1354–1361 (2020).

21. C. P. Kelleher, A. Wang, G. I. Guerrero-García, A. D. Hollingsworth, R. E. Guerra, B. J. Krishnatreya, D. G. Grier, V. N. Manoharan, P. M. Chaikin, Charged hydrophobic colloids at an oil–aqueous phase interface. *Phys. Rev. E* **92**, 062306 (2015).

22. E. M. Furst, Directing colloidal assembly at fluid interfaces. *Proc. Natl. Acad. Sci. U.S.A.* **108**, 20853–20854 (2011).

23. S. Razavi, B. Lin, K. Y. C. Lee, R. S. Tu, I. Kretzschmar, Impact of surface amphiphilicity on the interfacial behavior of janus particle layers under compression. *Langmuir* **35**, 15813–15824 (2019).

24. K. Kim, K. Park, G. Kim, H. Kim, M. C. Choi, S. Q. Choi, Surface charge regulation of carboxyl terminated polystyrene latex particles and their interactions at the oil/water interface. *Langmuir* **30**, 12164–12170 (2014).

25. R. McGorty, J. Fung, D. Kaz, V. N. Manoharan, Colloidal self-assembly at an interface. *Mater. Today* **13**, 34–42 (2010).

26. S. Srivastava, N. A. Kotov, Nanoparticle assembly for 1D and 2D ordered structures. *Soft Matter* **5**, 1146 (2009).

27. J. P. Pantina, E. M. Furst, Colloidal aggregate micromechanics in the presence of divalent ions. *Langmuir* **22**, 5282–5288 (2006).

28. B. J. Park, E. M. Furst, Micromechanics of colloidal aggregates at the oil–water interface. *Soft Matter* **7**, 7683 (2011).

29. G. A. Duncan, M. A. Bevan, Tunable aggregation by competing biomolecular interactions. *Langmuir* **30**, 15253–15260 (2014).

30. M. A. Gharbi, M. Nobili, M. In, G. Prévot, P. Galatola, J. B. Fournier, C. Blanc, Behavior of colloidal particles at a nematic liquid crystal interface. *Soft Matter* **7**, 1467–1471 (2011).

31. W.-S. Wei, M. A. Gharbi, M. A. Lohr, T. Still, M. D. Gratale, T. C. Lubensky, K. J. Stebe, A. G. Yodh, Dynamics of ordered colloidal particle monolayers at nematic liquid crystal interfaces. *Soft Matter* **12**, 4715–4724 (2016).

32. G. M. Koenig, I. H. Lin, N. L. Abbott, Chemoresponsive assemblies of microparticles at liquid crystalline interfaces. *Proc. Natl. Acad. Sci. U.S.A.* **107**, 3998–4003 (2010).

33. I. I. Smalyukh, S. Chernyshuk, B. I. Lev, A. B. Nych, U. Ognysta, V. G. Nazarenko, O. D. Lavrentovich, Ordered droplet structures at the liquid crystal surface and elastic-capillary colloidal interactions. *Phys. Rev. Lett.* **93**, 117801 117801 (2004).

34. F. Mukherjee, A. Shi, X. Wang, F. You, N. L. Abbott, Liquid crystals as multifunctional interfaces for trapping and characterizing colloidal microplastics. *Small* **19**, e2207802 (2023).

35. I.-H. Lin, G. M. Koenig, J. J. de Pablo, N. L. Abbott, Ordering of solid microparticles at liquid crystal─water interfaces. *J. Phys. Chem. B* **112**, 16552–16558 (2008).

36. J.-C. Loudet, P. Barois, P. Poulin, Colloidal ordering from phase separation in a liquid-crystalline continuous phase. *Nature* **407**, 611–613 (2000).

37. P. Poulin, H. Stark, T. C. Lubensky, D. A. Weitz, Novel colloidal interactions in anisotropic fluids. *Science* **275**, 1770–1773 (1997).

38. A. B. Nych, U. M. Ognysta, V. M. Pergamenshchik, B. I. Lev, V. G. Nazarenko, I. Muševič, M. Škarabot, O. D. Lavrentovich, Coexistence of two colloidal crystals at the nematic-liquid-crystal-air interface. *Phys. Rev. Lett.* **98**, 057801 (2007).

39. G. M. Koenig, R. Ong, A. D. Cortes, J. A. Moreno-Razo, J. J. de Pablo, N. L. Abbott, Single nanoparticle tracking reveals influence of chemical functionality of nanoparticles on local ordering of liquid crystals and nanoparticle diffusion coefficients. *Nano Lett.* **9**, 2794–2801 (2009).

40. N. Wang, J. S. Evans, C. Li, V. M. Pergamenshchik, I. I. Smalyukh, S. He, Controlled multistep self-assembling of colloidal droplets at a nematic liquid crystal–air interface. *Phys. Rev. Lett.* **123**, 087801 (2019).

41. T. Yamamoto, M. Yoshida, Self-assembled pseudo-hexagonal structures of colloidal particles at air–liquid crystal interface. *Appl. Phys. Express* **2**, 101501 (2009).

42. V. M. Pergamenshchik, Strong collective attraction in colloidal clusters on a liquid-air interface. *Phys. Rev. E* **79**, 011407 (2009).

43. D. Abras, G. Pranami, N. L. Abbott, The mobilities of micro- and nano-particles at interfaces of nematic liquid crystals. *Soft Matter* **8**, 2026–2035 (2012).

44. J. A. Moreno-Razo, E. J. Sambriski, G. M. Koenig, E. Díaz-Herrera, N. L. Abbott, J. J. de Pablo, Effects of anchoring strength on the diffusivity of nanoparticles in model liquid-crystalline fluids. *Soft Matter* **7**, 6828 (2011).

45. Y. Xu, Q. Ou, X. Wang, F. Hou, P. Li, J. P. van der Hoek, G. Liu, Assessing the mass concentration of microplastics and nanoplastics in wastewater treatment plants by pyrolysis gas chromatography–mass spectrometry. *Environ. Sci. Technol.* **57**, 3114–3123 (2023).

46. S. Zhao, M. Danley, J. E. Ward, D. Li, T. J. Mincer, An approach for extraction, characterization and quantitation of microplastic in natural marine snow using Raman microscopy. *Anal. Methods* **9**, 1470–1478 (2017).

47. E. M. Herzig, K. A. White, A. B. Schofield, W. C. K. Poon, P. S. Clegg, Bicontinuous emulsions stabilized solely by colloidal particles. *Nat. Mater.* **6**, 966–971 (2007).

48. K. Stratford, R. Adhikari, I. Pagonabarraga, J.-C. Desplat, M. E. Cates, Colloidal jamming at interfaces: A route to fluid-bicontinuous gels. *Science* **309**, 2198–2201 (2005).

49. J. Deng, W. Dong, R. Socher, L.-J. Li, K. Li, L. Fei-Fei, "ImageNet: A large-scale hierarchical image database," in *2009 IEEE Conference on Computer Vision and Pattern Recognition* (IEEE, 2009), pp. 248–255.

50. A. Chattopadhay, A. Sarkar, P. Howlader, V. N. Balasubramanian, "Grad-CAM++: Generalized gradient-based visual explanations for deep convolutional networks," in *2018 IEEE Winter Conference on Applications of Computer Vision (WACV)* (IEEE, 2018), pp. 839–847.

51. L. M. Flores-Tandy, A. V. García-Monjaraz, E. A. van Nierop, E. A. Vázquez-Martínez, J. Ruiz-Garcia, S. Mejía-Rosales, Fractal aggregates formed by ellipsoidal colloidal particles at the air/water interface. *Colloids Surf. A Physicochem. Eng. Asp.* **590**, 124477 (2020).

52. R. Yang, K. Bernardino, X. Xiao, W. R. Gomes, D. A. Mattoso, N. A. Kotov, P. Bogdan, A. F. de Moura, Graph theoretical description of phase transitions in complex multiscale phases with supramolecular assemblies. *Adv. Sci.* **11**, e2402464 (2024).

53. D. Reker, Y. Rybakova, A. R. Kirtane, R. Cao, J. W. Yang, N. Navamajiti, A. Gardner, R. M. Zhang, T. Esfandiary, J. L'Heureux, T. von Erlach, E. M. Smekalova, D. Leboeuf, K. Hess, A. Lopes, J. Rogner, J. Collins, S. M. Tamang, K. Ishida, P. Chamberlain, D. Yun, A. Lytton-Jean, C. K. Soule, J. H. Cheah, A. M. Hayward, R. Langer, G. Traverso, Computationally guided high-throughput design of self-assembling drug nanoparticles. *Nat. Nanotechnol.* **16**, 725–733 (2021).

54. G. Bae, T. Park, I.-H. Song, Surface modification of polymethylmethacrylate (PMMA) by ultraviolet (UV) irradiation and IPA rinsing. *Micromachines* **13**, 1952 (2022).

55. H. van der Wel, J. Lub, Surface modification of polymethylmethacrylate by UV light as studied by TOF-SIMS. *Surf. Interface Anal.* **20**, 373–378 (1993).

56. Y. Sun, J. Yuan, T. Zhou, Y. Zhao, F. Yu, J. Ma, Laboratory simulation of microplastics weathering and its adsorption behaviors in an aqueous environment: A systematic review. *Environ. Pollut.* **265**, 114864 (2020).

57. K. G. de Castro Monsores, A. O. da Silva, S. de Sant' Ana Oliveira, R. P. Weber, P. F. Filho, S. N. Monteiro, Influence of ultraviolet radiation on polystyrene. *J. Mater. Res. Technol.* **13**, 359–365 (2021).

58. C. Li, B. Jiang, J. Guo, C. Sun, C. Shi, S. Huang, W. Liu, C. Wu, Y. Zhang, Aging process of microplastics in the aquatic environments: Aging pathway, characteristic change, compound effect, and environmentally persistent free radicals formation. *Water* **14**, 3515 (2022).

59. B. Q. Kim, T. Ren, A. Majumder, D. Lee, Nanoconfinement-induced shift in photooxidative degradation pathway of polystyrene. *J. Colloid Interface Sci.* **683**, 841–847 (2025).

60. S. S. Alavian Petroody, S. H. Hashemi, L. Škrlep, B. Mušič, C. A. M. van Gestel, A. Sever Škapin, UV light causes structural changes in microplastics exposed in bio-solids. *Polymers* **15**, 4322 (2023).

61. J. Yang, L. Cang, Q. Sun, G. Dong, S. T. Ata-Ul-Karim, D. Zhou, Effects of soil environmental factors and UV aging on Cu2+ adsorption on microplastics. *Environ. Sci. Pollut. Res.* **26**, 23027–23036 (2019).

62. J. Lin, D. Yan, J. Fu, Y. Chen, H. Ou, Ultraviolet-C and vacuum ultraviolet inducing surface degradation of microplastics. *Water Res.* **186**, 116360 (2020).

63. Q. Wang, X. Wangjin, Y. Zhang, N. Wang, Y. Wang, G. Meng, Y. Chen, The toxicity of virgin and UV-aged PVC microplastics on the growth of freshwater algae chlamydomonas reinhardtii. *Sci. Total Environ.* **749**, 141603 (2020).

64. H. Walker, Stability of particle flocs upon addition of natural organic matter under quiescent conditions. *Water Res.* **35**, 875–882 (2001).

65. M. Bandekar, F. Abdolahpur Monikh, J. Kekäläinen, T. Tahvanainen, R. Kortet, P. Zhang, Z. Guo, J. Akkanen, J. T. T. Leskinen, M. A. Gomez-Gonzalez, G. Krishna Darbha, H.-P. Grossart, E. Valsami-Jones, J. V. K. Kukkonen, Submicron plastic adsorption by peat, accumulation in sphagnum mosses and influence on bacterial communities in peatland ecosystems. *Environ. Sci. Technol.* **56**, 15661–15671 (2022).

66. M. Zark, T. Dittmar, Universal molecular structures in natural dissolved organic matter. *Nat. Commun.* **9**, 3178 (2018).

67. E. L. Sharp, S. A. Parsons, B. Jefferson, Seasonal variations in natural organic matter and its impact on coagulation in water treatment. *Sci. Total Environ.* **363**, 183–194 (2006).

68. L. Pontoni, V. Roviello, M. Race, L. Savignano, E. D. van Hullebusch, G. Esposito, F. Pirozzi, M. Fabbricino, Supramolecular aggregation of colloidal natural organic matter masks priority pollutants released in water from peat soil. *Environ. Res.* **195**, 110761 (2021).

69. J. C. Everts, B. Senyuk, H. Mundoor, M. Ravnik, I. I. Smalyukh, Anisotropic electrostatic screening of charged colloids in nematic solvents. *Sci. Adv.* **7**, (2021).

70. H. Mundoor, B. Senyuk, M. Almansouri, S. Park, B. Fleury, I. I. Smalyukh, Electrostatically controlled surface boundary conditions in nematic liquid crystals and colloids. *Sci. Adv.* **5**, eaax4257 (2019).

71. R. R. Shah, N. L. Abbott, Coupling of the orientations of liquid crystals to electrical double layers formed by the dissociation of surface-immobilized salts. *J. Phys. Chem. B* **105**, 4936–4950 (2001).

72. E. J. W. Verwey, Theory of the stability of lyophobic colloids. *J. Phys. Chem.* **51**, 631–636 (1947).

73. L. Di Michele, F. Varrato, J. Kotar, S. H. Nathan, G. Foffi, E. Eiser, Multistep kinetic self-assembly of DNA-coated colloids. *Nat. Commun.* **4**, 2007 (2013).

74. L. Lin, J. Zhang, X. Peng, Z. Wu, A. C. H. Coughlan, Z. Mao, M. A. Bevan, Y. Zheng, Opto-thermophoretic assembly of colloidal matter. *Sci. Adv.* **3**, e1700458 (2017).

75. Y.-Y. Luk, K.-L. Yang, K. Cadwell, N. L. Abbott, Deciphering the interactions between liquid crystals and chemically functionalized surfaces: Role of hydrogen bonding on orientations of liquid crystals. *Surf. Sci.* **570**, 43–56 (2004).

76. J. M. Brake, M. K. Daschner, Y. Y. Luk, N. L. Abbott, Biomolecular interactions at phospholipid-decorated surfaces of liquid crystals. *Science* **302**, 2094–2097 (2003).

77. M. Dijkstra, E. Luijten, From predictive modelling to machine learning and reverse engineering of colloidal self-assembly. *Nat. Mater.* **20**, 762–773 (2021).

78. S. Tammina, Transfer learning using VGG-16 with deep convolutional neural network for classifying images. *Int. J. Sci. Res. Publ.*. **9**, 143–150 (2019).

79. R. L. Kumar, J. Kakarla, B. V. Isunuri, M. Singh, Multi-class brain tumor classification using residual network and global average pooling. *Multimed. Tools Appl.* **80**, 13429–13438 (2021).

80. A. Kirillov, E. Mintun, N. Ravi, H. Mao, C. Rolland, L. Gustafson, T. Xiao, S. Whitehead, A. C. Berg, W.-Y. Lo, P. Dollár, R. Girshick, "Segment anything," in *2023 IEEE/CVF International Conference on Computer Vision (ICCV)* (IEEE, 2023), pp. 3992–4003.

81. F. H. Frimmel, Characterization of natural organic matter as major constituents in aquatic systems. *J. Contam. Hydrol.* **35**, 201–216 (1998).

82. J. Yan, R. Manelski, B. Vasilas, Y. Jin, Mobile colloidal organic carbon: An underestimated carbon pool in global carbon cycles? *Front. Environ. Sci.* **6**, 148 (2018).

83. A. Philippe, G. E. Schaumann, Interactions of dissolved organic matter with natural and engineered inorganic colloids: A review. *Environ. Sci. Technol.* **48**, 8946–8962 (2014).

84. M. Maghsoodi, C. Jacquin, B. Teychené, G. Lesage, S. D. Snow, Delineating the effects of molecular and colloidal interactions of dissolved organic matter on titania photocatalysis. *Langmuir* **39**, 3752–3761 (2023).

85. L. Torrisi, A. M. Roszkowska, L. Silipigni, M. Cutroneo, A. Torrisi, Effects of 365 nm UV lamp irradiation of polymethylmethacrylate (PMMA). *Radiat. Eff. Defects Solids* **179**, 264–274 (2024).
